# T-cell lymphoma-associated STAT3 variants impose a type 1 regulatory-like phenotype

**DOI:** 10.3389/fimmu.2026.1726565

**Published:** 2026-05-05

**Authors:** Aaron B. Schultz, Molly Dalzell, Luis Nivelo, Sarah E. Henrickson, Alejandro V. Villarino

**Affiliations:** 1 Department of Microbiology and Immunology, Miller School of Medicine, University of Miami, Miami, FL, United States; 2 Sylvester Comprehensive Cancer Center, University of Miami, Miami, FL, United States; 3 Division of Allergy and Immunology, Department of Pediatrics, Children’s Hospital of Philadelphia, Philadelphia, PA, United States; 4 Institute for Immunology and Immune Health, University of Pennsylvania Perelman School of Medicine, Philadelphia, PA, United States; 5 Department of Microbiology, Perelman School of Medicine, University of Pennsylvania, Philadelphia, PA, United States

**Keywords:** cytokine, IL-10, IL-27, JAK-STAT, leukemia, lymphoma, STAT3, T cell

## Abstract

STAT3 signaling is fundamental to T cells, where it underlies basic cellular processes like metabolism and apoptosis, as well as specialized processes like effector differentiation and cytokine production. However, mutations of STAT3 are strikingly prevalent in T-cell cancers, and aberrant or excessive STAT3 signaling is thought to mobilize cellular pathways that encourage malignancy. To better understand how STAT3 mutations drive T-cell cancers, we compared two frequent cancer-associated variants, Y640F and N647I, at the cellular and molecular levels. Using a retrogenic system, we demonstrate that they are qualitatively similar yet quantitatively distinct; each bears a gain-of-function phenotype, but Y640F has greater transcriptome-wide effects. We also discovered that these and other common STAT3 mutants invoke a T regulatory 1 (Tr1) gene program characterized by expression of IL-10 and other factors that dampen T-cell responses, including LAG3 and CD39. Importantly, “Tr1 skewing” is evident in both mouse T cells expressing cancer-associated STAT3 variants and humans afflicted with T-cell malignancies. These studies advance current understanding of how cancer-associated mutations impact STAT3 function and reveal anti-inflammatory properties that may help transformed T cells persist, expand, and/or avoid eradication.

## Introduction

STAT3 signaling is fundamental to immune cells, and notably, both gain-of-function (GOF) and loss-of-function (LOF) STAT3 mutations manifest severe immunological disorders (1–4). Thus, STAT3 has been extensively studied in lymphocytes, specifically T and B cells, where it participates in fundamental cellular processes like metabolism and apoptosis and in specialized immunological processes like differentiation and cytokine production (5, 6). As with all STAT family members, STAT3 is a signal-dependent transcription factor subject to upstream signaling cascades. Typically, these begin with extracellular cytokines binding transmembrane receptors, which, in turn, trigger cytoplasmic Janus kinases (JAKs) or receptor tyrosine kinases (RTKs) to phosphorylate STAT3 at tyrosine 705. Phospho-STAT3 then dimerizes, translocates to the nucleus, and distributes throughout the genome via consensus DNA motifs, thereby modulating gene transcription (7–9).

**Figure 1:**
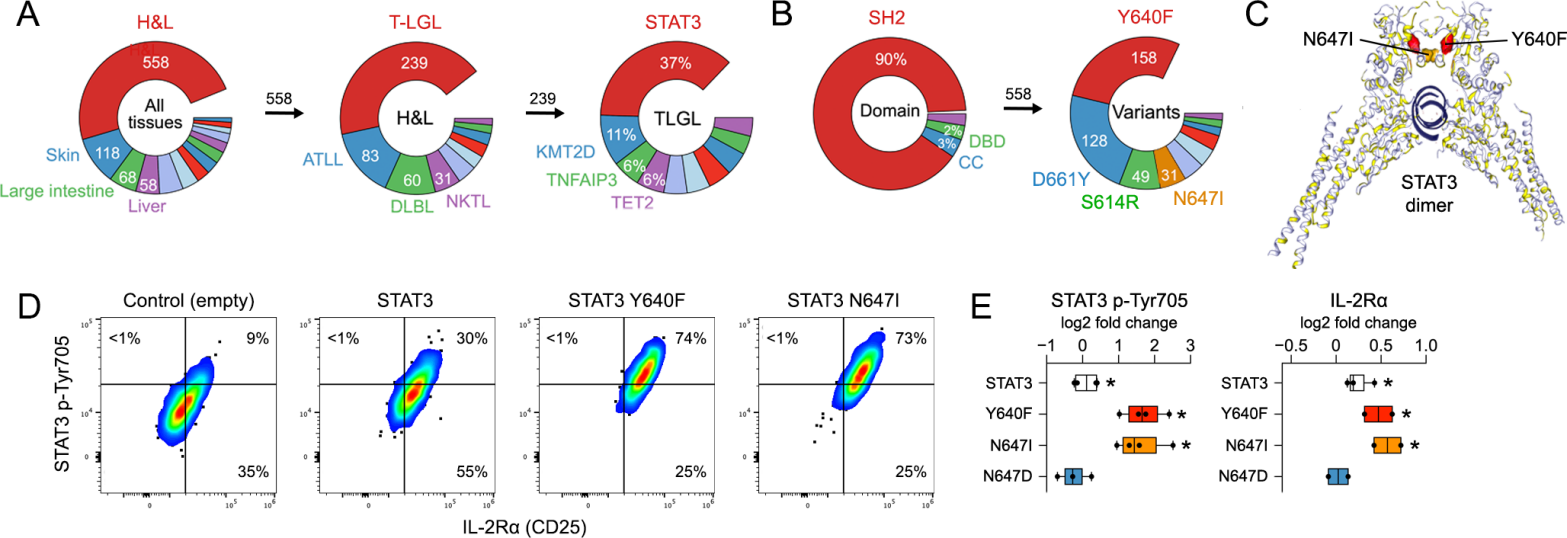
STAT3 SH2 variants are linked to T-cell cancers. **(A, B)** Donut plots tally COSMIC entries (patients) bearing single-nucleotide, missense STAT3 variants. Full, annotated variant roster presented in **Supplementary Table 1**. **(A)** The left donut shows variants broken down by tissue. The middle shows hematopoietic and lymphoid (H&L) malignancies broken down by subtype. The right shows prevalence of STAT3 variants in T-LGLL. **(B)** The left shows H&L STAT3 variants broken down by protein domain. The right shows common SH2 variants. **(C)** The ribbon model shows STAT3 homodimer bound to DNA, highlighting Y640 and N647 at the dimer interface. **(D, E)** *Stat3^−/−^* T cells were transduced with control (empty) or STAT3 retroviruses, then cultured with IL-27, and assayed by cytometry. **(D)** Pseudocolor plots show IL-2Rα (CD25) and p-STAT3 in CD4^+^ T cells. **(E)** Box plots compile mean fluorescence intensity values from CD4^+^ T cells. Stars denote *p <*0.05 relative to empty vector control. Cytometry experiments include at least three biological replicates assayed over at least two experiments. Replicates and statistical tests detailed in **Supplementary Table 2**.

STAT3 activity is a cardinal feature of immune cell cancers (10–12). In fact, STAT3 activity is not only present but also considered a “tier 1” driver of disease, per COSMIC (13). Furthermore, somatic mutations in *STAT3* are seen in ~5% of all hematological malignancies and are conspicuously evident in T-cell cancers, particularly T-cell large granular lymphocytic leukemias (T-LGLLs), which bear *STAT3* mutations at a rate of ~40% (**Figure 1A**) (14–21). Given its driver status, STAT3 has become a coveted drug target (22–28), creating a pressing need to understand downstream oncogenic properties. Moreover, despite clear genetic links, it remains unclear why T-cell cancers are the most likely outcome for STAT3 mutations, why some manifest T-LGLL, while others manifest alternative T-cell cancers, like adult T-cell leukemia (ATL), or even more perplexing, why some manifest either T-LGLL or alternative cancers.

STAT3 is activated downstream of many cytokines, hormones, and growth factors. Among these, cytokines operating via the GP130 co-receptor are keenly relevant for T cell malignancies (7, 26, 29, 30). IL-6, the prototype GP130 family member, has long been viewed as a cancer driver due to its ability to foster T-cell proliferation, and its influence on T-cell subsets that promote or subvert antitumor inflammation (10, 11, 29–31). Specifically, IL-6 limits antitumor Th1-type responses and encourages pro-tumor Th17-type responses (32). IL-27 is another relevant GP130 family member that fosters T-cell proliferation (30) and promotes hematopoietic malignancy through tumor-intrinsic effects (33, 34). However, unlike IL-6, IL-27 enhances antitumor immunity by encouraging Th1 and limiting Th17 differentiation (32), and is required for optimal tumoricidal CD8^+^ T-cell responses (35).

Strikingly, >90% of STAT3 mutations linked to blood cancers occur within the SH2 domain, which mediates receptor docking and dimerization (**Figure 1B**). The most frequent and, fittingly, most studied is Y640F, a single-nucleotide variant that converts amino acid 640 from tyrosine to phenylalanine (**Figure 1B**; **Supplementary Figure S1**, **Supplementary Table S1**). Prior work has shown that Y640F bears a GOF phenotype with enhanced activation (17, 19, 36–41), dimerization (40) and mobilization of downstream target genes linked to hematopoietic malignancy, including SBNO2 and 4-1BB (41, 42). The next most frequent variants are D661Y and D661V (collectively), followed by S614R and N647I (**Figure 1B**; **Supplementary Figure S1**, **Supplementary Table S1**). Available data point to similarities between these and Y640F—each also bears a GOF phenotype - but downstream effects are far less appreciated and head-to-head comparisons are mostly lacking (17, 19, 39–41, 43).

To better understand how STAT3 drives T-cell malignancy, we directly compared Y640F and N647I, which are located adjacently within the SH2 domain (**Figure 1C**). Using a retrogenic system, we determined that they are qualitatively similar yet quantitatively distinct, with Y640F exhibiting greater transcriptome-wide effects. We also discovered that these and other STAT3 SH2 mutants potently induce a T regulatory 1 (Tr1) gene program characterized by the immuno-regulatory cytokine, IL-10, and other factors known to dampen T-cell responses, most notably LAG3 and CD39. Importantly, Tr1 skewing is evident in both mouse T cells artificially expressing STAT3 SH2 mutants and within the native repertoire of humans with T-LGLL or ATL. Together, these studies advance basic understanding of how SH2 mutations alter STAT3 activity and reveal anti-inflammatory properties that may contribute to immune evasion by T-cell cancers.

## Results

### N647I is a gain-of-function STAT3 variant

To study cancer-associated variants, we devised a retrogenic model system. Briefly, STAT3-deficient CD4^+^ and CD8^+^ T cells were transduced with retroviral vectors expressing either control STAT3 or cancer-associated variants, such that the only available STAT3 was the one that we introduced. Wild-type T cells were also run in parallel as these approximate the scenario in patients, where normal and mutant STAT3 alleles coexist. Transduced cells were then cultured with IL-27 for 48 h and inspected by flow cytometry or RNA-seq. IL-27 was the chosen stimulus because it is a potent STAT3 activator linked to hematological malignancies and because both receptor components, GP130 and IL-27R, are highly expressed in both CD4^+^ and CD8^+^ T cells (44). Y640F was the chosen benchmark variant because it is the most frequent and best understood, while N647I was the chosen test variant because it is also found within the SH2 domain, in fact, adjacent to the dimer interface (**Figure 1C**). Moreover, while not extensively studied, N647I is among the four most common STAT3 variants, which together account for >75% of all hematological malignancies bearing mutant STAT3 (**Figure 1B**; **Supplementary Figure S1**, **Supplementary Table S1**). N647D, a loss-of-function variant linked to hyper-IgE syndrome (43), was also included because it impacts the same amino acid residue as N647I. Importantly, different genotypes (*Stat3^+/+^* and *Stat3^−/−^*) and variants were cultured in parallel at similar densities with shared reagents, including a single IL-27 cocktail that was distributed across samples. Cytometry confirmed efficient transductions, with 30%–50% of cells appearing GFP positive after 48 h (**Supplementary Figure S2A**), and RNA-seq confirmed robust expression of *Stat3* transcripts, with both control and variant vectors outputting more than the endogenous *Stat3* locus in WT cells (**Supplementary Figure S2B**). Also, despite evidence that STAT3 plays a role in T regulatory cells (45), neither Y640F nor N647I induced FOXP3, the lineage-defining transcription factor for this subset. More than 2% of cells expressed FOXP3 after 72 h in culture, the standard experimental endpoint, whether transduced with control or mutant STAT3 (**Supplementary Figure S2C**). This was true downstream of IL-6, IL-21, or IL-27 (**Supplementary Figure S2C**) and was confirmed at the transcript level; FOXP3 was undetectable by RNA-seq (not shown).

**Figure 2:**
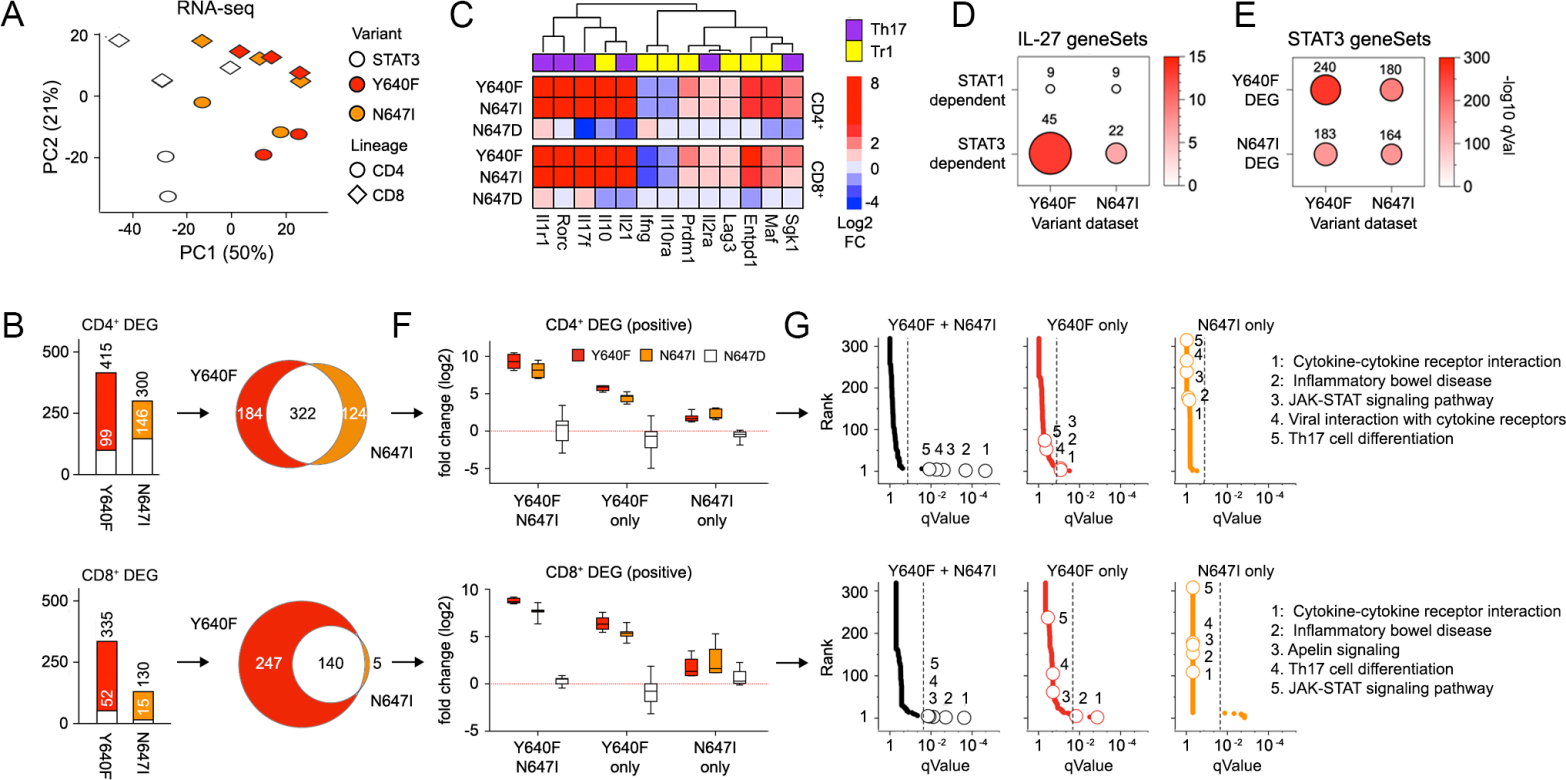
STAT3 N647I is a gain-of-function variant. (**A–G**) *Stat3^−/−^* T cells were transduced with control (empty) or STAT3 retroviruses, cultured with IL-27, then sorted and assayed by RNA-seq. **(A)** Scatter plot shows PCA results. **(B)** Bar plots tally DEG called relative to “normal” STAT3 in CD4^+^ (top) or CD8^+^ T cells (bottom). The lower section denotes negatively regulated DEG. Positively (black) and negatively (white) regulated DEG counts are shown. Venn plots compare DEG called in CD4^+^ (top) or CD8^+^ T cells (bottom) transduced with Y640F or N647I. **(C)** The heatmap shows log2-transformed fold-change values for select DEGs. **(D, E)** Scatter plot shows hypergeometric testing of positive DEGs from CD4^+^ T cells against **(D)** STAT1- or STAT3-dependent genes induced by IL-27 in CD4^+^ T cells, or **(E)** genes positively regulated by Y640F or N647I in CD4^+^ T cells. Size proportional to the number of DEG hits; total shown. Color shading proportional to *q* value (FDR). All gene sets detailed in **Supplementary Table 3** and **Supplementary Table 4**. **(F, G)** DEGs were categorized based on whether mobilized by Y640F, N647I, or both (per adjacent Venn plots). **(F)** Box plots compile log2-transformed, fold-change values for each category. **(G)** Positively regulated DEGs from each category were subjected to hypergeometric testing against the KEGG database. Rankline plots show *p*-values and *p*-value ranks for all pathways, with select pathways and ranks noted. Full datasets reported in **Supplementary Table 8**. RNA-seq experiments include at least two biological replicates assayed over at least two experiments. Cytometry experiments include at least three biological replicates assayed over at least two experiments. Replicates and statistical tests detailed in **Supplementary Table 2**.

To gauge STAT3 activity, we measured phosphorylation of tyrosine 705, the key instigating event for STAT3 signaling, and induction of IL-2Rα (CD25), a known downstream target gene (46). Consistent with prior reports (17, 19, 39–41), we found that both Y640F and N647I triggered more p-STAT3 and CD25 than control STAT3 (**Figures 1D, E**). This hyperactivity was evident in both CD4^+^ and CD8^+^ T cells and in both STAT3-sufficient and STAT3-deficient backgrounds (**Figures 1D, E**; **Supplementary Figures S2D, E**). For deeper insights, we next compared transcriptomes. Principal component analysis (PCA) showed clear separation between control STAT3 and variants, with the latter clustering tightly together regardless of lineage or genotype (**Figure 2A**; **Supplementary Figure S3A**). Next, we identified differentially expressed genes (DEGs) relative to control STAT3 and found that Y640F and N647I were comparable in CD4^+^ T cells but not in CD8^+^ T cells, where Y640F had a stronger effect (**Figure 2B**). Both variants mainly induced transcripts (i.e., positive DEG > negative DEG), including known STAT3 target genes like SOCS3 and IL-21 (**Figures 2B, C**; **Supplementary Figures S3B, C**). By contrast, N647D did not induce pSTAT3 or IL-2Rα (**Figures 1D, E**) and did not mobilize downstream transcripts (**Figure 2C**). Pathway analysis further highlights analogy between Y640F and N647I; both DEG sets were highly enriched for STAT3 target genes (**Figure 2D**) and scored highly when cross-referenced against one another (**Figure 2E**). Thus, transcriptome-wide analysis affirms commonalities between STAT3 variants but also points to lineage-restricted differences, with Y640F appearing more potent in CD8^+^ T cells.

Next, we directly compared the Y640F and N647I DEG sets. First, we noted that most DEGs were shared in CD4^+^ T cells and that, in both CD4^+^ and CD8^+^ T cells, few were mobilized by N647I that were not mobilized by Y640F (**Figure 2B**). However, it was obvious that Y640F was more active in CD8^+^ T cells, reflected in 2-fold more “Y640F only” DEGs than “shared” DEGs (**Figure 2B**). To explore this disparity, we compared effect size (i.e., transcript fold-change values) for DEG classified as “shared,” “Y640F only,” or “N647I only” and found that those mobilized by both Y640F and N647I were most labile (i.e., had the greatest fold-change values), followed by “Y640F only” and then “N647I only” (**Figure 2F**). We also noted that “Y640F only” DEGs were still responsive to N647I (and vice versa). By necessity, each DEG set was mutually exclusive, so we interpret that the variance across biological replicates likely masked some commonality between Y640F and N647I that was still captured by fold-change values. To follow up, we ran pathway analysis and learned that, by far, “shared” DEGs bore the most enriched pathways, including several known to be regulated by STAT3, like JAK–STAT signaling and Th17 differentiation (**Figure 2G**). The “Y640F only” set also bore some enrichment, albeit orders of magnitude less than the “shared set,” and the “N647I only” set bore almost none (**Figure 2G**). Together, these data argue that Y640F and N647I are mostly similar—they mobilize many of the same DEGs and, in turn, influence many of the same cellular processes. Few genes appear strictly subject to one variant or the other, and those that do fail to coalesce into biological pathways.

To address lineage-restricted effects, we compared DEG sets from CD4^+^ and CD8^+^ T cells. A shared transcriptional program was immediately evident for Y640F; most DEGs mobilized in CD4^+^ T cells were also mobilized in CD8^+^ T cells, and vice versa (**Supplementary Figure S3D**). The same was true for N647I, although it mobilized 3-fold more DEGs in CD4^+^ T cells than CD8^+^ T cells (**Supplementary Figure S3D**). Also, DEGs called in both CD4^+^ and CD8^+^ T cells were more labile than those called in either lineage alone and carried the most enrichment for STAT3-regulated pathways (**Supplementary Figures S3E, F**). Reminiscent of Y640F versus N647I (**Figure 2F**), DEGs classified as “CD4 only” were still mobilized in CD8^+^ T cells (and vice versa), indicating that some commonality was masked by variance across replicates (**Supplementary Figure S3E**). Therefore, transcriptional responses downstream of STAT3 SH2 variants appear largely stereotypical, similar across both variants and T-cell lineages.

### STAT3 SH2 variants impose a Tr1 phenotype

**Figure 3:**
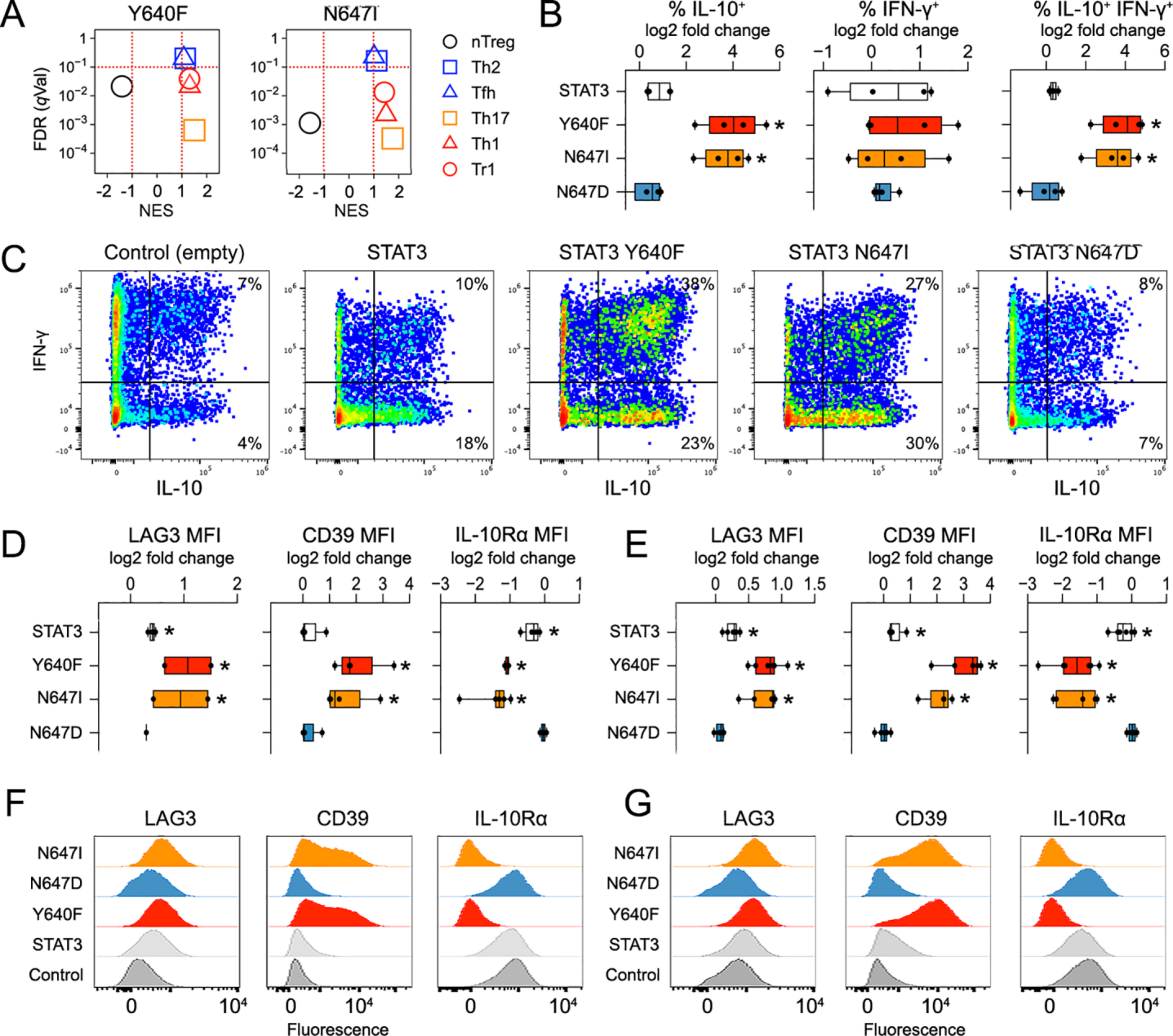
STAT3 N647I imposes a Tr1 phenotype. **(A)** *Stat3^−/−^* CD4^+^ T cells were transduced, cultured with IL-27, and assayed by RNA-seq. Transcripts were then ranked based on pairwise comparisons to “normal” STAT3 and subjected to GSEA against gene sets defining the indicated T-cell subsets (**Supplementary Table 3**). Scatter plots show normalized enrichment score versus *q* value (FDR). **(B–G)** *Stat3^−/−^* T cells were transduced, cultured with IL-27, and assayed by cytometry. **(B)** Box plots compile frequencies of IL-10^+^ and/or IFN-γ^++^ cells. **(C)** Pseudocolor plots show representative intracellular cytokine measurements. **(D, E)** Box plots compile mean fluorescence intensity values for LAG3, CD39, and IL-10Rα in **(D)** CD4^+^ or **(E)** CD8^+^ T cells. **(F, G)** Histograms show representative surface marker measurements in **(F)** CD4^+^ or **(G)** CD8^+^ T cells. RNA-seq experiments include at least two biological replicates assayed over at least two experiments. Cytometry experiments include at least three biological replicates assayed over at least two experiments. Stars denote *p <*0.05 relative to empty vector control. Replicates and statistical tests detailed in **Supplementary Table 2**.

Given their hyperactive nature, we reasoned that Y640F and N647I may skew CD4^+^ T-cell differentiation toward subsets known to be reliant on STAT3 (32, 47–50). Following this thread, we mined our RNA-seq datasets to determine if, relative to control STAT3, they are better able to mobilize genes associated with Th17-, Tr1-, and Tfh-type effectors. Gene set enrichment analysis showed that, indeed, this is the case with Th17 registering as the most enriched gene set (**Figure 3A**). Tr1 was also highly enriched, but surprisingly, Tfh was not despite evidence that STAT3 supports Tfh programming (**Figure 3A**) (47). Treg responses were depressed (**Figure 3A**), in line with studies showing that STAT3 signaling limits induction of Treg from naive precursors (51, 52).

Tr1-type responses potently suppress antitumor immunity (53, 54). Thus, we explored Tr1 programming as a potential immune evasion strategy downstream of STAT3 SH2 variants. To start, we determined if Tr1-defining genes are hyperresponsive and found that, indeed, they are. Relative to control STAT3, Y640F and N647I were each better able to induce transcription of *Il10*, the emblematic Tr1 cytokine; *Maf*, a key Tr1-specifying transcription factor; and other elements of the Tr1 program like the inhibitory receptor, *Lag3*, and the ectonucleotidase, *Entpd1* (CD39) (**Supplementary Figure S3G**) (53, 55). Next, we measured downstream proteins and the results were impressive on all counts. Y640F and N647I were each far better than the control STAT3 at inducing IL-10, LAG3, and CD39, regardless of genotype or lineage (**Figures 3B–G**; **Supplementary Figure S4A**). Follow-up experiments revealed three other salient features about this Tr1-inducing capacity: 1) it was blunted in WT cells, suggesting both dimerization and competition between mutant and non-mutant STAT3 (**Supplementary Figures 4B, C**); 2) it was evident under Th1 and Th17 polarizing conditions (**Supplementary Figure S5A**), suggesting that it may be relevant across inflammatory settings; and 3) it was evident downstream of multiple GP130 family cytokines: IL-6, which has long been implicated in hematological malignancies (26), and L-27, which has more recently been implicated (44) and is the principal STAT3 stimulus used in our studies (**Supplementary Figure S5B**). Also, noteworthy, the expression of IL-10Rα was strongly inhibited by both variants, suggesting a negative feedback loop whereby STAT3 limits IL-10-driven STAT3 activation (**Figures 3D–G**; **Supplementary Figure S4A**). Taken together, these data certify that Y640F and N647I each invoke a Tr1 phenotype and suggest that this property may be broadly relevant.

**Figure 4:**
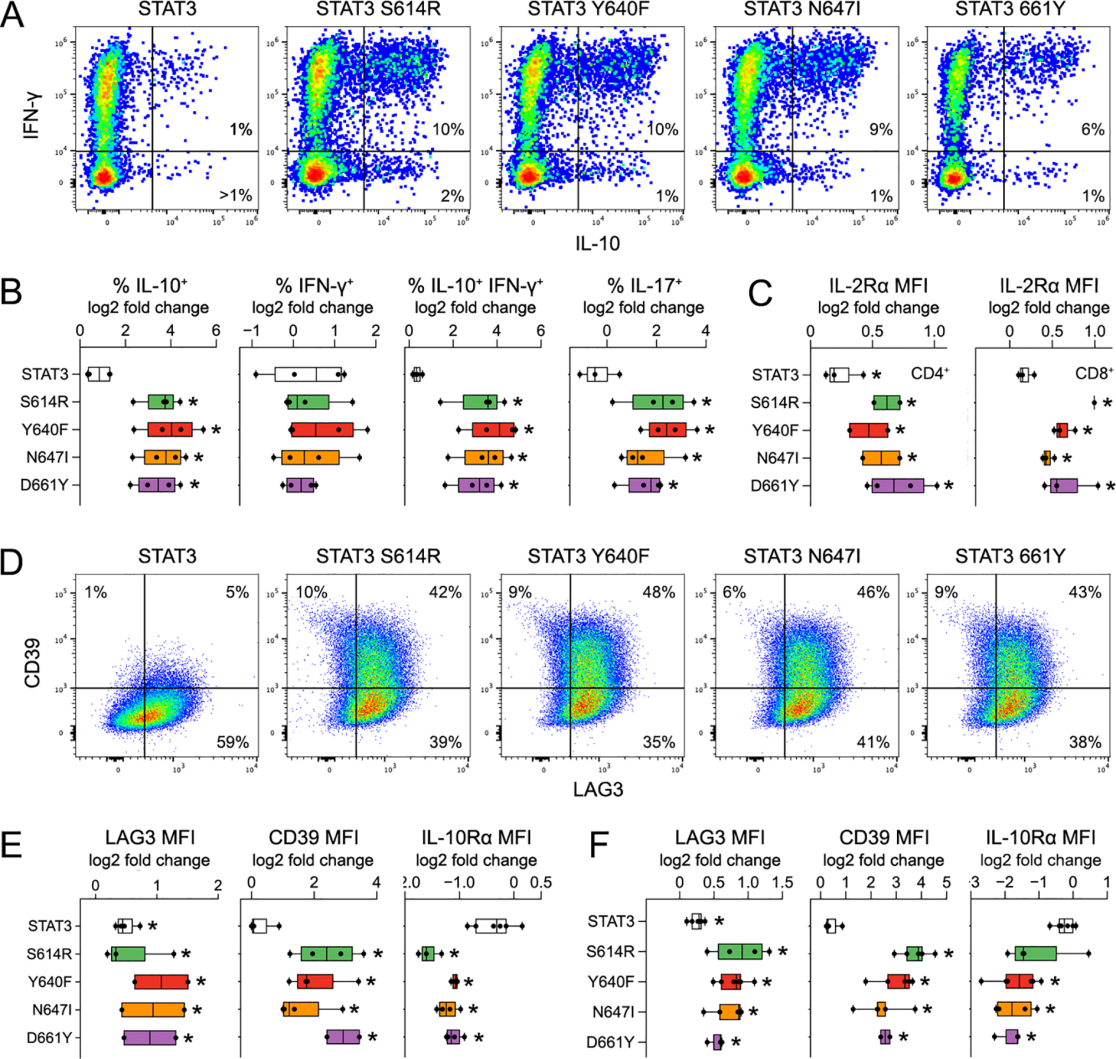
Tr1 skewing is a common feature of STAT3 SH2 variants. (**A–F**) *Stat3^−/−^* T cells were transduced with control (empty) or STAT3 retroviruses, then cultured with IL-27, and assayed by cytometry. **(A)** Pseudocolor plots show representative IL-10 and IFN-γ^+^ measurements in CD4^+^ T cells. Frequencies of single or double positive cells noted. **(B)** Box plots compile frequencies of single or double positive cells. **(C, D)** Histograms show representative LAG3, CD39, and IL-10Rα measurements in **(C)** CD4^+^ or **(D)** CD8^+^ T cells. **(E, F)**. Box plots compile mean fluorescence intensity values in **(E)** CD4^+^ or **(F)** CD8^+^ T cells. Cytometry experiments include at least three biological replicates assayed over at least two experiments. Stars denote *p <*0.05 relative to empty vector control. Replicates and statistical tests detailed in **Supplementary Table 2**.

To determine if Tr1 skewing is a universal feature of STAT3 SH2 variants, we tested S614R and D661Y, respectively, which were the third and second most frequent variants among hematopoietic malignancies (**Figure 1B**; **Supplementary Figure S1**). Again, the results were clear and impressive. Like Y640F and N647I, S614R and D661Y each potently induced IL-10, LAG3, and CD39, regardless of genotype or lineage (**Figures 4A–F**; **Supplementary Figures S6A–C**). They also suppressed IL-10Rα, again, indicating a negative feedback loop whereby the STAT3 SH2 variant impels IL-10 production but also limits its autocrine effects (**Figures 4E, F**, **Supplementary Figure S6C**).

**Figure 5:**
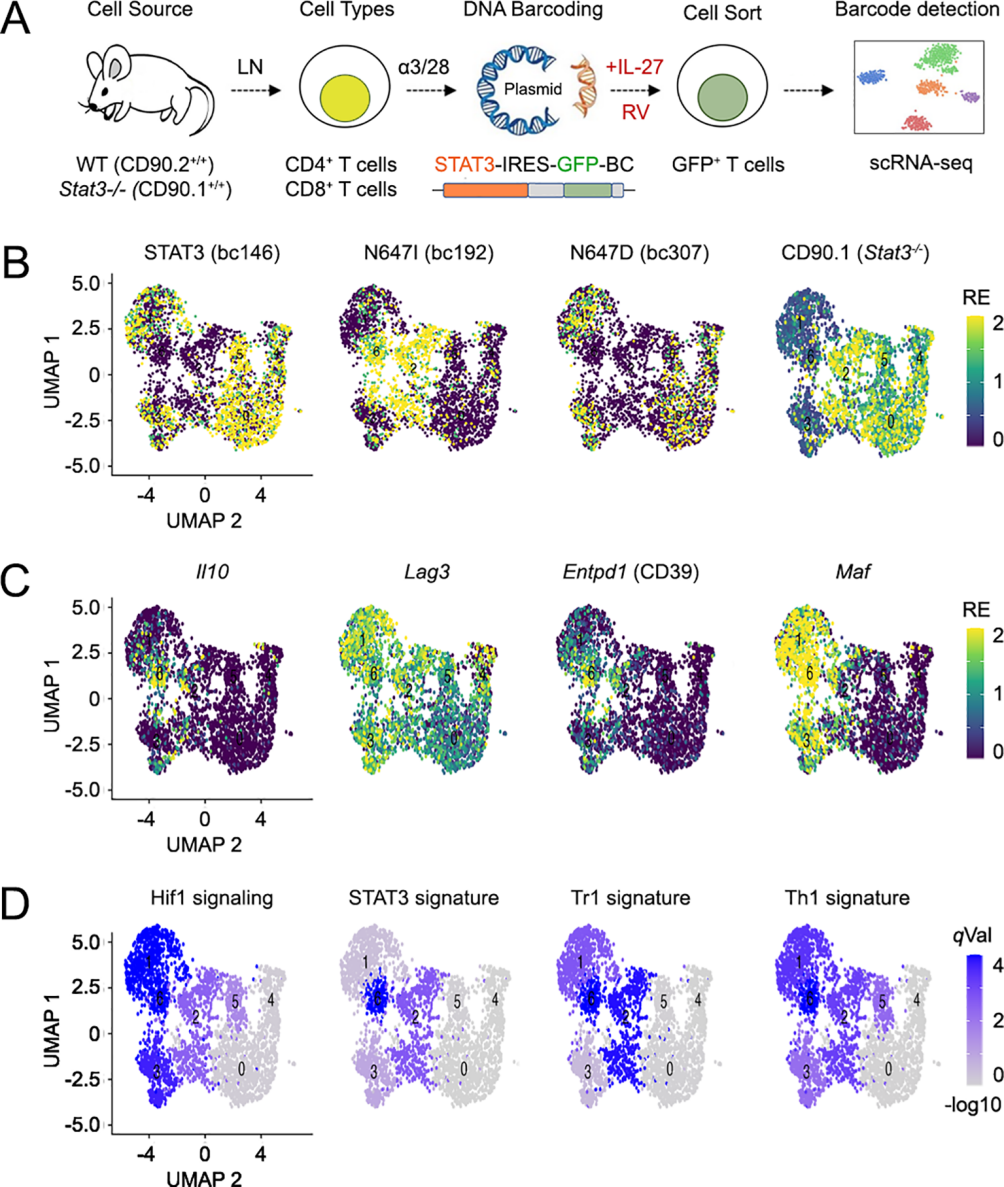
STAT3 N647I mobilizes a Tr1 gene program. **(A)** The cartoon illustrates key features of our retrogenic model adapted for scRNA-seq endpoint. Briefly, T cells are transduced with retroviral vectors expressing DNA-tagged STAT3, cultured with IL-27, and processed for scRNA-seq. DNA tags are then used to determine which vector, or variant, each cell contains. Mouse and plasmid line drawings originally published by the Public Library of Science and edited and used in accordance Wikimedia Creative Commons Attribution 2.5 Generic license. **(B–D)** CD4^+^ T cells bearing >1 exclusive tags (i.e., multi-tagged cells) were selected per **Supplementary Figure 7**. **(B)** Feature plots show the distribution of tags used to distinguish STAT3 variants and the congenic marker used to distinguish *Stat3^+/+^* (CD90.1^low^) and *Stat3^−/−^* (CD90.1^high^) cells. **(C)** Feature plots show the relative expression of Tr1-associated genes. **(D)** Feature plots show hypergeometric testing results against the indicated KEGG or custom gene sets. All gene sets detailed in **Supplementary Table 3**. Replicates and statistical tests detailed in **Supplementary Table 2**.

Cytometry showed that STAT3 SH2 variants can invoke the classic IL-10^+^ IFN-γ^++^ Tr1 phenotype (**Figure 4A**). However, we also found that sometimes IL-10 was not co-expressed with IFN-γ^+^ and/or other Tr1 markers, and often it was not expressed at all (**Figure 4A**). To explore this heterogeneity, we adapted our retrogenic system for scRNA-seq analysis, focusing on N647I with N647D and reference STAT3 serving as controls. First, DNA barcodes were appended to relevant plasmid vectors, such that transduced cells would be “tagged” with a permanent record of which vector they incorporated. Next, vectors were pooled and packaged into retrovirus, which was then used to transduce primary T cells, which, in turn, were cultured with IL-27, sorted, and processed for scRNA-seq (**Figure 5A**). Importantly, by leveraging congenic markers, STAT3-deficient cells were sequenced together with WT cells (**Figures 5B–D**). CD4^+^ and CD8^+^ T cells were also sequenced together and then distinguished by standard means (**Supplementary Figures S7A–C**).

DNA tags were detected in approximately half of the sequenced cells (**Supplementary Figure S7B**). This was surprising because all cells were sorted based on GFP expression, which is directly linked to the DNA tags. We interpret that many tags were not detected due to a “dropout” problem inherent to droplet-based scRNA-seq, rather than deficiencies in our experimental system and/or analysis pipelines (56). Notwithstanding, we were encouraged that no cells bearing more than one tag type were detected (not shown) and proceeded by focusing on those bearing at least two exclusive tags (i.e., “multi-tagged cells”; **Supplementary Figure S7D**). Crucially, uniform manifold approximation and projection (UMAP) analysis showed clear separation between CD4^+^ T cells expressing N647I and those expressing N647D or control STAT3, the latter tending to congregate together (**Figure 5B**; **Supplementary Figure S8A**). Furthermore, a nexus of Tr1 genes, including *Il10*, *Maf*, *Lag3*, and *Entpd1* (CD39), was plainly evident in sections of the UMAP populated by N647I-transduced cells (**Figure 5C**; **Supplementary Figure S8B**). Pathway analysis affirmed the enrichment of a Tr1 gene signature, along with Th1, STAT3, and hypoxia signatures (**Figure 5D**). These effects were seen in both STAT3-sufficient and STAT3-deficient backgrounds (**Supplementary Figure S8B**) and in both CD4^+^ and CD8^+^ cells (**Figure 5C**; **Supplementary Figure S9**), indicating a stereotypical response.

As with our cytometry studies, we noticed that only a portion of the UMAP populated by N647I-transduced cells bore a Tr1 phenotype. In fact, most relevant areas were largely devoid of Tr1 markers (**Figure 5C**). Thus, while it holds true that N647I can invoke the Tr1 program, other signals must be needed for full implementation, which only a subset of cells received at the point of assay. To further explore this heterogeneity, we subset STAT3-deficient, N647I-transduced CD4^+^ cells and ran a new UMAP analysis, which revealed five transcriptionally distinct clusters (**Supplementary Figure S10A**). Based on emblematic Tr1 markers like *Il10*, *Maf*, *Lag3*, *Entpd1*, and *Prdm1*, we determined that Tr1 cells were concentrated in cluster 3, with a few scattered in cluster 4 (**Supplementary Figures S10A–C**). Accordingly, cluster 3 was highly enriched for HIF1α signaling, a pathway that we and others have linked to the Tr1 program (**Figure 5D**; **Supplementary Figure S10D**) (57). By contrast, clusters 1 and 2 were each highly enriched for cell cycle-associated genes and MYC targets, suggesting that they were actively proliferating and, perhaps, had not yet enacted an effector program (**Supplementary Figure S10D**). Cluster 0 was highly enriched for interferon-γ^+^ signaling, suggesting that these cells may have skewed more toward Th1 than Tr1 programming (**Supplementary Figure S10D**).

**Figure 6:**
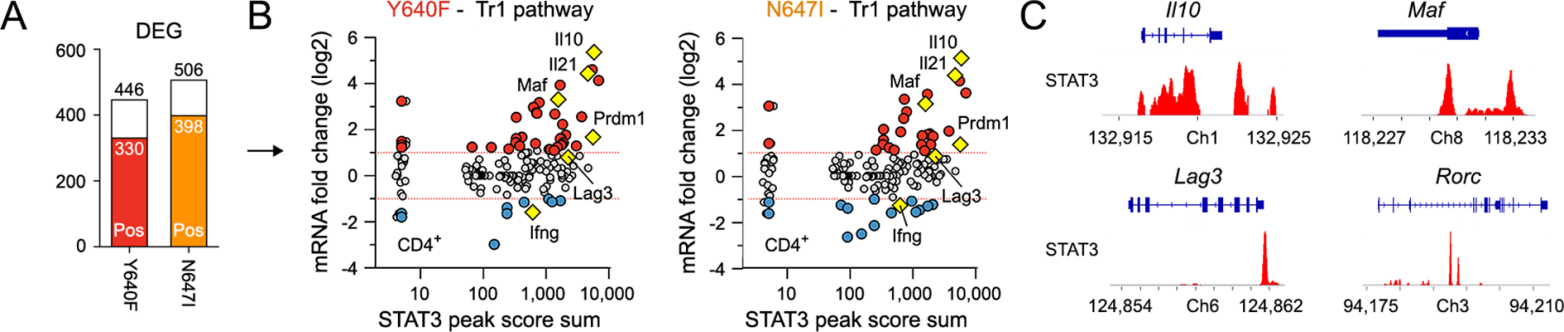
STAT3 localizes to Tr1-associated gene loci. **(A)** CD4^+^ T-cell DEGs (from **Figure 2B**) were cross-referenced with STAT3 ChIP-seq peaks captured in CD4^+^ T cells pulsed with IL-27. Bar plot shows the proportion of DEGs proximally bound by STAT3 (colored section). Total DEGs (top) and STAT3-bound DEGs (middle) are indicated. Full, annotated peak set presented in **Supplementary Table 9**. **(B)** Scatter plots show cumulative STAT3 peak amplitude and transcript fold change (normal versus variant STAT3) for Tr1-associated genes. Tr1 gene set detailed in **Supplementary Table 3**. Dotted red lines denote 2-fold change. **(C)** Genome browser tracks show STAT3 localization at the *Il10*, *Maf*, *Lag3*, and *Rorc* loci. For ChIP-seq, two biological replicates were analyzed per group with similar results. Only one is shown, per Hirahara et al.

STAT family transcription factors control gene expression, in large part, by acting as classical transcription factors; they find and engage target genes via sequence motifs found within DNA regulatory elements that instruct transcription, usually promoters and enhancers (8, 9). To confirm that STAT3 localizes to Tr1-associated gene loci, we mined a STAT3 ChIP-seq dataset captured in CD4^+^ T cells stimulated with IL-27, similar to our culture conditions (32). First, we cross-referenced with our transcriptome datasets and learned that, indeed, STAT3 binds near to most DEGs mobilized by Y640F or N647I in CD4^+^ T cells (**Figure 6A**). Importantly, this includes emblematic Tr1 genes that are hyperresponsive to these and other SH2 variants, such as *Il10*, *Maf*, and *Lag3* (**Figures 6B, C**). STAT3 was also found near many Th17-associated genes, in line with the idea that it is crucial for this subset (**Supplementary Figure S11**). Thus, we can infer that Tr1 skewing by STAT3 SH2 mutants is tied to direct effects on transcription of Tr1-associated genes.

### Evidence for Tr1 skewing in T-cell malignancies

STAT3 SH2 variants are strongly linked to T-cell malignancies. Thus, we hypothesized that afflicted individuals may bear evidence of Tr1 skewing. To explore this idea, we next mined single-cell RNA-seq datasets captured in patients suffering from T-LGLL or ATLL, the most prevalent cancers among STAT3 SH2 variants (**Figure 1A**). T-cell receptor (TCR) sequencing data were available for the T-LGLL dataset, so we focused on hyperexpanded T-cell clones, per Huuhtanen et al. (**Supplementary Figures S12A, B**) (58). TCR sequencing data were not available for the ATLL dataset, so, instead, we considered the entire CD3ϵ^+^ T-cell compartment (**Supplementary Figures S12C, D**) (59). Of note, both T-LGLL and ATLL are characterized by the accumulation of hyperexpanded T clones (60, 61).

**Figure 7:**
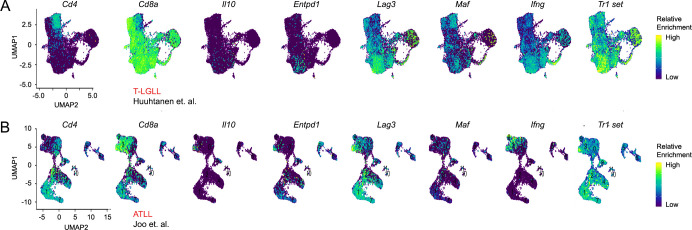
T-cell cancers bear evidence of Tr1 skewing. **(A, B)** scRNA-seq was performed on **(A)** T-LGLL and **(B)** ATLL blood samples per Huuhtanen et al. and Joo et al., respectively. Feature plots show relative enrichment of *Cd4*, *Cd8a*, and Tr1-associated genes and the Tr1 signature (detailed in **Supplementary Table 3**). scRNA-seq analysis further detailed in **Supplementary Figure 12**.

CD8^+^ T cells dominated both the T-LGLL and ATLL datasets (**Figures 7A, B**), consistent with prior work showing that they are principal mediators of these diseases (62, 63). Crucially, both datasets also bore evidence of Tr1 skewing in the form of cells expressing IL-10, CD39, LAG3, and MAF (**Figures 7A, B**). Some of these markers were more broadly expressed than others: IL-10 and CD39 were on the low end of the spectrum, while LAG3 and MAF were on the high end. We interpret that the relative paucity of IL-10^+^ and CD39^+^ cells is due to a lack of acute stimulation. Unlike our *in vitro* system, where cells coincidently received strong TCR, CD28, and STAT3 stimuli, the T-LGLL and ATLL datasets were captured *ex vivo* and, thus, are asynchronous; some cells may have recently encountered STAT3 stimuli, but most likely had not. Another notable finding was that, when present, Tr1-type cells were found in areas populated by CD8^+^ T cells. This was surprising because the Tr1 phenotype is mainly associated with CD4^+^ T cells (53) and, given that CD8^+^ T cells are hyperexpanded, suggests that a large pool of Tr1-type cells may be present in T-LGLL and ATLL patients. Thus, *post hoc* scRNA-seq analysis affirms that T-cell malignancies associated with STAT3 SH2 variants bear evidence of Tr1 skewing, specifically within the hyperexpanded CD8^+^ T-cell compartment that is thought to drive pathology.

## Discussion

Somatic mutations within the SH2 domain of STAT3 are strongly linked to T-cell malignancies, most notably T-LGLL and ATLL (64). However, it remains unclear how these mutations unleash latent oncogenic potential and, in turn, why T-cell malignancies are the most likely outcome. Furthermore, >80 STAT3 variants have been linked to lymphoid cancers, yet only a handful have been studied in any detail, typically in a one-by-one manner. Here, we address these gaps by comparing Y640F, the most frequent and well-understood SH2 variant, to N647I, another common SH2 variant that has not been extensively studied. Using a retrogenic system, we determined that they are qualitatively similar—able to mobilize a shared suite of target genes—but quantitatively distinct, with Y640F appearing more potent, particularly in CD8^+^ T cells. These findings are in line with prior work showing that, similar to other STAT3 SH2 variants, Y640F and N647I each bear a GOF phenotype with enhanced dimerization and DNA-binding capabilities (17, 19, 39–43). Also consistent with prior work (41, 42), we find that this GOF phenotype manifests across the transcriptome, leading to enhanced mobilization of downstream pathways that promote oncogenesis and/or suppress antitumor immunity. Conspicuous among these is the T regulatory 1 (Tr1) differentiation program characterized by induction of IL-10, a potent anti-inflammatory cytokine; MAF, a key lineage-defining transcription factor; and immune checkpoints LAG3 and CD39. In fact, we establish that “Tr1 skewing” is a universal feature of STAT3 SH2 mutants. D661Y and S614R, respectively, the second and third most frequent cancer-associated variants, also exhibit this property. Given the anti-inflammatory nature of Tr1 cells (53, 55), we now propose that Tr1 skewing is a mechanism of immune evasion for T-cell malignancies, as it is for solid cancers (53, 54), and that IL-10, LAG3, CD39, and other elements of the Tr1 program are potential therapeutic targets. However, we must also recognize that this assertion is based on *in vitro* studies with non-transformed T cells exposed to a single stimulus and that we have not shown that Tr1 skewing affects bystander T-cell responses. Thus, further *in vivo* studies are needed to establish functional consequences and, in turn, clinical relevance. For instance, it will be critical to determine if a Tr1 phenotype is seen in ectopic or genetic models of T-cell lymphoma, and whether blocking Tr1 products (e.g., IL-10, CD39) can ameliorate disease. Fundamentally, it remains possible that Tr1 skewing does not play a major role in T-cell malignancies and that other properties of STAT3, like its ability to promote T-cell stemness (65, 66), are more relevant in this setting.

Why STAT3 SH2 mutants are so adept at mobilizing the Tr1 program is an intriguing open question. It is understood that STAT3 normally invokes the Tr1 program by localizing to and promoting transcription of IL-10 and Tr1-associated genes (48, 49, 67, 68). Thus, one interpretation of our findings is that SH2 mutants do exactly what STAT3 does only: due to enhanced dimerization, phosphorylation, and/or DNA binding, they do it better. This is certainly plausible and, given that STAT3 hyperactivity is seen across T-cell cancers, may account for phenotypic similarities between patients with and without STAT3 mutations (62, 69, 70). Therefore, building on this latter point, we now propose that Tr1 skewing is a universal rather than mutant-specific feature of T-cell malignancies. However, we must also acknowledge evidence that SH2 mutants behave differently from normal STAT3. First, and most compelling, transcriptional signatures differ between T-LGLL patients with and without STAT3 mutations, the latter typically bearing less pathology and responding better to treatment (14, 39, 42, 70, 71). Second, unlike SH2 variants, ectopic expression of normal STAT3 (i.e., just increasing total STAT3 content) did not enhance Tr1 skewing in WT T cells. Third, downstream responses for SH2 variants were blunted in WT T cells relative to STAT3-deficient counterparts, which suggests both dimerization and competition between normal and mutant STAT3. Considering that most individuals bearing somatic STAT3 mutations retain one normal STAT3 allele, this tempering effect may contribute to the slow, indolent nature of T-LGLL and other T-cell malignancies.

STAT3 SH2 mutants mobilize the Tr1 program in both CD4^+^ and CD8^+^ T cells. This is striking because Tr1 is not a view as a typical outcome for CD8^+^ T cells. In fact, we found few if any lineage-restricted effects for STAT3 SH2 mutants; most genes and pathways mobilized in CD8^+^ T cells were also mobilized in CD4^+^ T cells (and vice versa), and almost no pathways were mobilized exclusively in either lineage. Importantly, this includes STAT3-driven pathways that are known to promote tumor growth, including Th17 differentiation and hypoxia, and a newly identified negative feedback loop where STAT3 limits expression of IL-10Rα, thereby limiting autocrine, IL-10-driven STAT3 activation (50). As with Tr1 skewing, we hypothesize that increased elaboration of these and other STAT3-dependent pathways downstream of SH2 mutants contributes to the onset and/or progression of T-cell malignancies.

Considering that the CD8 compartment is typically more impacted than the CD4 compartment in T-cell malignancies, we were encouraged to find that Tr1 skewing was evident in CD8^+^ T cells from T-LGLL and ATL patients. However, only a small subset bore the full Tr1 program (i.e., co-expression of IL-10, MAF, LAG, CD39, and IFN-γ^+^). One explanation is that patient samples were not restimulated and, thus, were not synchronized like with our retrogenic system. It is also likely that signals beyond STAT3 are needed to complete Tr1 differentiation and that relatively few T-LGLL or ATL cells received them immediately prior to sequencing. Other factors known to promote Tr1 responses include T-cell receptor and STAT1 signaling pathways and the transcription factors IRF4, BHLHE40, NFIL3, and BLIMP1 (49, 67, 68, 72, 73). In fact, the same may be true *in vitro* where downstream responses to STAT3 SH2 variants were clearly heterogeneous. This was particularly evident in our scRNA-seq studies, which showed that only a subset of N647I-transduced cells achieved the full Tr1 program. As with patient samples, IL-10 and CD39 were limiting, while MAF and LAG3 were more broadly expressed. Also, noteworthy, N647I-expressing cells bearing an IL-10^neg^ CD39^neg^ phenotype were highly proliferative and did not express IFN-γ^+^, IL-4, IL-13, or IL-17A, suggesting that they had not yet committed to an effector fate. Nevertheless, our finding that STAT3 SH2 mutants are potent inducers of the Tr1 program is a critical insight, relevant not only to T-cell differentiation but also how STAT3 drives T-cell malignancies, ultimately pointing to previously unrecognized therapeutic avenues.

## Methods

### COSMIC database mining

STAT3 COSMIC entries were downloaded from https://cancer.sanger.ac.uk/cosmic on 25 January 2025 (13) and then subset based on the following criteria and annotations: single amino acid substitutions (1,151 total); primary tissue = hematopoietic and lymphoid (558 total); histology subtype 1 = T-cell large granular lymphocytic leukemia (239 total), adult T-cell lymphoma-leukemia (83 total) (**Figure 1A****;** **Supplementary Table 1**). A ribbon model of the STAT3 homodimer with highlighted Y640 and N647 residues was rendered with COSMIC 3D (https://cancer.sanger.ac.uk/cosmic3d) (**Figure 1C**) (74).

### Animals

*Stat3* floxed mice were generated as described (75). These were backcrossed to C57BL/6J (>10 generations) and then further crossed with *Cd4*-Cre mice (strain: 022071 from Jackson Labs, USA) to generate *Stat3* ^flox/flox^ *Cd4*-Cre^+/−^ mice, which lack STAT3 selectively in T cells (referred to as *Stat3^−/−^* mice). Wild-type controls were *Cd4*-Cre*^−/−^* littermates or C57BL/6J purchased from Jackson Labs (strain: 000664). Both male and female mice were used. All experiment cohorts were sex- and age-matched. Animals were housed and handled in accordance with federal, state, and university guidelines, and all experiments were approved by the University Animal Care and Use Committee. Animals were humanely euthanized per NIH guidelines by carbon dioxide asphyxiation (30%–70% chamber volume per minute) followed by cervical dislocation.

### STAT3 variant constructs

Control mouse *Stat3* cDNA was synthesized based on a reference sequence for the most abundant mRNA isoform (ENSMUST00000127638.8) and then cloned into an MoMLV-based plasmid vector (MigR1) immediately upstream of a dual internal ribosome entry sequence (IRES) and GFP cassette (synthesis and cloning by GenScript). STAT3 variants were generated by site-directed mutagenesis of control STAT3 plasmid based on COSMIC annotations: S614R = c.1842C>G, Y640F = c.1919A>T, N647I = c.1940A>T, N647D = c.1939A>G, D661Y = c.1981G>T (mutagenesis by GenScript). For scRNA-seq studies, 17 nucleotide DNA “tags” were synthesized as single strands (Integrated DNA Technologies, Coralville, Iowa) and then annealed and cloned into control STAT3, STAT3 N647I, or STAT3 N647D plasmids immediately downstream of GFP (Control = bc146 = TGTTCGGTACGCAATCC; N647I = bc192 = GGACAACGGTACGATGG; N647D = bc307 = GTTACCGCCGTTGTGGA). For retroviral packaging, STAT3 plasmids and pCL-Eco “helper” plasmid were co-transfected into 293T cells (ATCC, Manassas, VA, USA) using Lipofectamine (Invitrogen, Carlsbad, CA, USA), and then virus-containing supernatants were collected 48 h later.

### T-cell purification, culture, and transduction

Mesenteric (mLN) and peripheral lymph nodes (pLN; inguinal, brachial, axillary, and superficial cervical) were dissected from 8- to 16-week-old mice and processed to single-cell suspensions by mechanical dissociation through 70-μM cell strainers. Suspensions (0.5 × 10^6^ cells/mL) were then stimulated with plate-bound anti-CD3ϵ (10 μg/mL; clone: 17A2; BioXcell, Lebanon, NH, USA) and anti-CD28 (10 μg/mL; clone 37.51; BioXcell, Manassas, VA, USA) in the presence of blocking anti-mouse IL-4 and anti-mouse IFN-γ^+^ (10 μg/mL each; clones 11B11 and XMG1.2; BioXcell, Manassas, VA, USA). Twenty-four hours later, cultures were exposed to viral supernatant for 1 h (centrifuged at 2,200 rpm, 18°C) and then further cultured for 48 h in the presence of mouse IL-27 (10 ng/mL; R&D Systems, Minneapolis, MN, USA), anti-mouse IL-4, and anti-mouse IFN-γ^+^. All cultures were maintained in RPMI-1640 medium supplemented with 10% fetal calf serum, 1% sodium pyruvate, 1% non-essential amino acids, 10 mM of HEPES, 0.1% β-mercaptoethanol, 100 U/mL of penicillin, and 100 mg/mL of streptomycin.

### Cytometry

For surface antigens, cells were stained and washed in phosphate-buffered saline supplemented with 0.5% bovine serum albumin and 0.1% sodium azide. For phospho-STAT3, cells were first pulsed with 10 ng/mL of IL-27 for 1 h, then fixed with 2% formaldehyde, permeabilized with 100% methanol, and stained with fluorochrome-labeled anti-human/mouse pY703 STAT3 (clone 4/P-STAT3; BD Biosciences, San Jose, CA, USA) together with fluorochrome-labeled antibodies directed at surface markers. For cytokines, cultures were first pulsed with phorbol 12-myristate 13-acetate (PMA; 50 ng/mL; Sigma, USA) and ionomycin (500 ng/mL; Sigma, USA) for 4 hours plus brefeldin A for the final 2 h (BFA; 10 μg/mL) and then fixed and permeabilized with Cytofix/Cytoperm (BD Biosciences, San Jose, CA, USA). Fluorochrome-labeled antibodies were purchased from Thermo-Fisher, BD Biosciences, or BioLegend. Dead cells were excluded using Live/Dead Aqua (Invitrogen, Carlsbad, CA, USA) or Zombie NIR (BioLegend, San Diego, CA, USA). Biological replicates and statistical tests for all experiments are detailed in **Supplementary Table 2**.

### Bulk RNA-seq

Viable, GFP^+^CD4^+^CD8α^neg^ or GFP^+^CD4^neg^CD8α^+^ T cells were sorted 48 h post-transduction (>95% purity). Two to three biological replicates were collected for each experimental group with similar numbers of cells per replicate (20–100 × 10^3^ per replicate). These were lysed in TRIzol reagent, and total RNA was purified by phenol–chloroform extraction with GlycoBlue as co-precipitant (7–15 μg per sample; Life Technologies, Carlsbad, CA, USA). Poly(A)^+^ mRNA was then enriched by oligo-dT-based magnetic separation, and single-end read libraries were prepared with NEBNext Ultra RNA Library Prep Kit (New England Biolabs, Ipswich, MA, USA). Sequencing was performed with NextSeq 2000 (Illumina, San Diego, CA, USA), then 50-bp reads were aligned to the mouse genome build mm10 with *tophat2* (20–50 × 10^6^ per sample), and assembled with *cufflinks*, and gene-level counts were compiled with *featureCounts*. To minimize normalization artifacts, genes failing to reach an empirically defined count threshold were purged using *htsfilter*. Genes (11–13 × 10^3^) were typically recovered post-filtering, regardless of genotype or experimental group. Counts were normalized and DEGs were identified by quasi-likelihood F testing using *edgeR*. DEG calls denote >2-fold pairwise changes and a Benjamini–Hochberg (BH) adjusted *p*-value <0.05. Transcripts per million (TPM) were compiled with *edgeR*. *clusterprofiler* was used for gene set enrichment analysis (GSEA) or hypergeometric testing (HGT) against the KEGG and Molecular Signatures database or custom gene sets (gene sets catalogued in **Supplementary Table 3**; usage detailed in **Supplementary Table S4**). Venn plots were rendered with *vennerable*, heatmaps with *pheatmap*, and all the other plots with *ggplot2* or *Datagraph* (Visual Data Tools Inc., Chapel Hill, NC, USA). *edgeR*-derived statistics, TPM values, and DEG calls are presented in **Supplementary Table S5**-**S7**. *clusterprofiler*-derived statistics and annotations are presented in **Supplementary Table S8**. Biological replicate counts for all experimental groups are detailed in **Supplementary Table S2**.

### ChIP-seq data mining

The STAT3 ChIP-seq dataset was generated from CD4^+^ T cells cultured *in vitro* with IL-27 as described (32) and downloaded from the NCBI Small Read Archive via GEO (GSE65621). Short reads were aligned using *bowtie* and then non-redundant reads were mapped to the mouse genome mm9 with *macs2* using default settings and “input” controls as reference for peak calling. *homer* was used to annotate peaks and test for DNA motif enrichment. Gene proximal peaks were defined as occurring within introns, exons, UTRs, or <20 kb of transcriptional start sites (**Supplementary Table S9**). Scatter plots were rendered with *Datagraph*, and genome browser tracks with *IGV*.

### Single-cell RNA-seq

WT and *Stat3^−/−^* lymphocytes were transduced and sorted as above, and then 10,000 viable GFP^+^ cells were sequenced using 10X Chromium Single Cell 3′ V3 chemistry on a NextSeq 2000 instrument. Quality control passing FASTQ reads were aligned to the reference genome mm10, assembled into transcripts, and packaged into cell-indexed, gene-level outputs using *cellranger count* (v7.0.0). These were then further analyzed with *seurat* (v.4.0.2) using default function parameters unless otherwise noted. Briefly, the experimental groups were first merged into a single dataset, then normalized and scaled using *seurat* sctTransform. Data points were excluded if they had >15% mitochondrial transcripts (dead/dying cells), >15,000 total transcripts or > 4,000 genes per cell (potential doublets, multiples), or <500 genes per cell (partial cells). UMAP reductions were then created using *seurat* RunPCA, *seurat* FindNeighbors, *seurat* FindClusters, and *seurat* RunUMAP, relying on the top 15 loadings for principal component analysis of the top 3,000 variable features. Final clustering resolution of 0.4 was chosen based on *clustree* analysis. DNA tags were detected and enumerated using custom R scripts based on Biddy et al. (76). Briefly, BAM outputs from *cellranger* were parsed using a custom gawk script to produce a sparse matrix linking DNA tags to 10× cell barcodes and UMI sequences for all captured cells. Next, “multi-tagged” cells bearing >1 unique DNA tag were subset (0.6 UMAP resolution; **Supplementary Figure S7D**), then further subset based on expression of CD4 (0.5 UMAP resolution) or CD8a (0.7 UMAP resolution) (**Figures 7C,D**). Feature plots and violin plots were rendered with *seurat*.

### scRNA-seq data mining

scRNA-seq data from T-LGLL patients (*n* = 11) and healthy donors (*n* = 6) were downloaded from ArrayExpress (accession E-MTAB-11170) and processed as above. See Huuhtanen et al. for details on patient genotypes and demographics (58). Published UMAP was then recreated, and hyperexpanded T-cell clones subset using embedded metadata and annotations (**Supplementary Figures S12A, B**). The ATLL scRNA-seq dataset GSE19574 is from the skin biopsy of a single 69-year-old male patient diagnosed but not yet treated for ATLL, according to Joo et al. (59). UMAP reduction was created as above (0.5 resolution), and then CD3ϵ^+^ T cells were subset and further analyzed at 0.5 UMAP resolution (**Supplementary Figures S12C, D**).

### Statistics and reproducibility

Biological and technical replicates for every experiment are detailed in **Supplementary Table 2**. All RNA-seq experiments include at least two biological replicates assayed over at least two experiments. All cytometry experiments include at least three biological replicates assayed over at least two experiments. Statistical variances and distributions were measured by paired *t*-test or Kolmogorov–Smirnov test as shown in **Supplementary Table 2**. Bonferroni correction was used to account for multiple testing in RNA-seq, ChIP-seq, and pathway analysis. Variance and multiple-testing corrections for scRNA-seq were performed using default *seurat* processes and parameters. When present, error bars denote standard deviation across >2 biological replicates.

## Data Availability

All data needed to evaluate the conclusions in the paper are present herein and/or the Supplementary Materials. Raw and processed sequencing data are uploaded to the NCBI GEO and SRA repositories under accession GSE328028. For bulk RNA-seq, Raw FASTQ data and gene-level transcript count tables were deposited. For scRNA-seq, raw FASTQ and processed data for DNA ‘tagged’ cells are deposited. All analysis pipelines and code are available upon request.

## Supplementary material

Supplementary Figure 1 Frequency and position of STAT3 SH2 variants. **(A)** Histogram shows position (x axis) and frequency (y axis) of single amino acid STAT3 variants linked to hematologic and lymphoid malignancies. Top 5 most frequent variants are noted. Each row represents a distinct variant, including deletions and nonsense variants. Full, annotated variant roster catalogued in **Supplementary Table 1**.

Supplementary Figure 2 Retrogenic system validation. **(A–D)** *Stat3*^+/+^ and *Stat3*^-/-^ lymphocytes were transduced with control or variant STAT3 vectors, then cultured with IL-27 and assayed by cytometry or RNA-seq. **(A)** Cytometry pseudocolor plots show GFP expression in transduced cells. **(B)** Box plots show STAT3 transcript counts (TPM) in transduced CD4^+^ (left) or CD8^+^ (right) T cells. **(C)** Cytometry pseudocolor plots show FOXP3 and IL-2Rα in transduced *Stat3*^-/-^ CD4^+^ T cells. **(D)** Cytometry histograms show p-STAT3 and IL-2Rα (CD25) in transduced *Stat3*^+/+^ and *Stat3*^-/-^CD8^+^ T cells.**(E**) Box plots compile mean fluorescence intensity values for *Stat3*^-/-^ CD8^+^ T cells. Stars denote p< 0.05 relative to empty vector control. Replicates and statistical tests detailed in **Supplementary Table 2**.

Supplementary Figure 3 Common and lineage-restricted effects of STAT3 SH2 variants. **(A–F)** *Stat3*^+/+^ and *Stat3*^-/-^ T cells were transduced with control or variant STAT3 vectors, cultured with IL-27, then processed for RNA-seq **(A)** Scatter plot shows PCA results for CD4^+^ T cells. **(B, C)** Volcano plots show variance and log2-transformed fold change values for all transcripts captured in *Stat3*^-/-^ CD4^+^ **(B)** or CD8^+^ **(C)** T cells. **(D)** Venn plots compare DEG mobilized by Y640F (top) or N647I (bottom) in CD4^+^ versus CD8^+^ T cells. **(E)** DEG were categorized based on whether mobilized in CD4^+^ T cells, CD8^+^ T cells or both (per adjacent Venn plots). Box plots compile log2-transformed, fold-change values for each category. **(F)** Positively regulated DEG from each category were subjected to hypergeometric testing against the KEGG database. Rankline plots shows adjusted p values and p value ranks for all pathways, with select pathways noted. **(G)** Scatter plots show transcript counts for Tr1-associated genes in CD4^+^ or CD8^+^ T cells. All genesets detailed in **Supplementary Tables 3, 4**. RNA-seq datasets further detailed in **Supplementary Tables 5–7**. Replicates and statistical tests detailed in **Supplementary Table 2**.

Supplementary Figure 4 Evidence for competition between normal STAT3 and SH2 variants. **(A–D)** *Stat3*^+/+^ and *Stat3*^-/-^ lymphocytes were transduced with control or variant STAT3 vectors, then cultured with IL-27 and assayed by cytometry. **(A)** Cytometry contour plots show surface protein measurements in CD4^+^ and CD8^+^ T cells. **(B)** Cytometry contour plots show percentage of CD39^high^ CD4^+^ and CD8^+^ T cells. **(C)** Box plots compile log2 fold change values for mean fluorescence intensities across cytometry experiments. Open boxes are *Stat3*^+/+^ cells, shaded boxes are *Stat3*^-/-^ cells. Stars denote significant differences across genotypes. Replicates and statistical tests detailed in **Supplementary Table 2**.

Supplementary Figure 5 STAT3 SH2 variants invoke Tr1 responses downstream of multiple cytokines. **(A, B)** *Stat3*^-/-^ lymphocytes were transduced with control or variant STAT3 vectors, then cultured and assayed by cytometry. **(A)** Contour plots show cytokine measurements for CD4^+^ T cells cultured under non-polarizing, Th1 polarizing or Th17 polarizing conditions. **(B)** Contour plots show surface protein measurements for CD4^+^ T cells cultured with IL-6. Box plots compile log2 fold change values for mean fluorescence intensities across cytometry experiments. Stars denote p < 0.05 relative to empty vector control. Replicates and statistical tests detailed in **Supplementary Table 2**.

Supplementary Figure 6 STAT3 SH2 variants impose a Tr1 phenotype in both CD4^+^ and CD8^+^ T cells. **(A-C)** *Stat3*^+/+^ and *Stat3*^-/-^ lymphocytes were transduced with control or variant STAT3 vectors, then cultured with IL-27 and assayed by cytometry. **(A)** Pseudocolor plots show LAG3 and CD39 measurements in *Stat3*^-/-^ CD8^+^ T cells. **(B)** Contour plots show LAG3 and CD39 measurements in *Stat3*^+/+^ CD4^+^ and CD8^+^ T cells. **(C)** Contour plots show LAG3 and IL-10R⍺ measurements in *Stat3*^-/-^ CD4^+^ and CD8^+^ T cells. Replicates and statistical tests detailed in **Supplementary Table 2**.

Supplementary Figure 7 Selection and analysis of barcode tagged T cells. **(A–C)** WT and *Stat3*^-/-^ T cells were transduced with barcoded control STAT3, STAT3 N647I or STAT3 N647D vectors, then cultured with IL-27 and assayed by scRNA-seq (as in **Figure 5A**). Featureplots show distribution of barcode tags for all cells. **(B)** Histograms stratify cells based on number of tags detected per cell (x axis). **(C)** Featureplots show distribution of *Cd4*, *Cd8a* and CD90.1, which marks *Stat3*^-/-^ cells, in cells bearing >1 exclusive tag (multi-tagged cells). **(D)** Histograms stratify cells based on the number of tags detected per cell (x axis). Only multi-tag selected cells shown.

Supplementary Figure 8 STAT3 N647I elicits a Tr1 program in CD4^+^ T cells. **(A, B)** scRNAseq was performed and multi-tagged cells subset as in **Supplementary Figure 7**. **(A)** Featureplots show UMAP clustering of *Cd4*^+^ cells broken down by DNA tags. **(B)** Violin plots show UMI counts for select Tr1-associated genes in *Cd4*^+^ cells, broken down by UMAP cluster (x axis) and genotype (color).

Supplementary Figure 9 STAT3 N647I elicits a Tr1 program in CD8^+^ T cells. **(A, B)** scRNAseq was performed and multi-tagged cells subset as in **Supplementary Figure 7**. **(A)** Featureplots show relative expression of variant-associated tags, CD90.1 and select Tr1-associated genes in *Cd8a*^+^ cells.

Supplementary Figure 10 Transcriptional heterogeneity in CD4^+^ T cells expressing N647I. **(A–D)** scRNAseq was performed and multi-tagged cells subset as in **Supplementary Figure 7**. **(A)** Left featureplot shows enrichment of DNA barcode 192, which marks cells transduced with N647I. Right featureplot shows UMAP clustering of *Cd4*^+^ CD90.1^+^ (*Stat3*^-/-^) barcode 192^+^ cells. **(B)** Featureplots show enrichment of markers representing each major UMAP cluster. **(C)** Scatter plots show relative enrichment (RE) and percent positive cells for select markers. **(D)** Top 200 positively regulated markers for each cluster were subjected to hypergeometric testing against the molecular signatures database. Rankline plots shows p values and p value ranks for all pathways, with select pathways and ranks noted.

Supplementary Figure 11 STAT3 localizes to Th17-associated gene loci. **(A)** CD4^+^ T cell DEG (Figure 2B) were cross-referenced with a STAT3 ChIP-seq dataset captured in CD4^+^ T cells pulsed with IL-27. **(A)** Scatter plots show cumulative STAT3 peak amplitude (x axis) and transcript fold change values (normal versus variant) for Th17-associated genes. Full, annotated STAT3 peak set presented in **Supplementary Table 9**. Th17 geneset detailed in **Supplementary Table 3**. Dotted red lines denote 2-fold change.

Supplementary Figure 12 scRNA-seq annotation and subsetting for T-LGLL and ATLL patient samples. scRNA-seq was performed on T-LGLL **(A, B)** and ATLL **(C, D)** blood samples per Huuhtanen et al. and Joo et al. **(A)** Featureplots show distribution of hyper-expanded T cells originating from T-LGLL patients or healthy controls. **(B)** Featureplots show distribution of hyper-expanded T cells from T-LGLL patients bearing mutant STAT3. **(C)** Featureplots show relative expression of *CD3E*, *CD4* and *CD8A* in all cells assayed from ATLL patients. **(D)** Featureplots show relative expression of *CD3E*, *CD4* and *CD8A* within the *CD3E*^+^ subset.

Supplementary Table 1 COSMIC STAT3 single amino acid variants

Supplementary Table 2 Replicate and statistics details

Supplementary Table 3 Gene set catalog

Supplementary Table 4 Gene set usage

Supplementary Table 5 RNA-seq pairwise statistics

Supplementary Table 6 RNA-seq TPM

Supplementary Table 7 RNA-seq DEG

Supplementary Table 8 RNA-seq KEGG pathway enrichment

Supplementary Table 9 Annotated STAT3 ChIPseq peaks
