# Supplementary figures and images for: T-cell lymphoma-associated STAT3 variants impose a type 1 regulatory-like phenotype
Source: Front Immunol. 2026 May 5;17:1726565. doi: 10.3389/fimmu.2026.1726565 (PMC13183623; doi:10.3389/fimmu.2026.1726565)

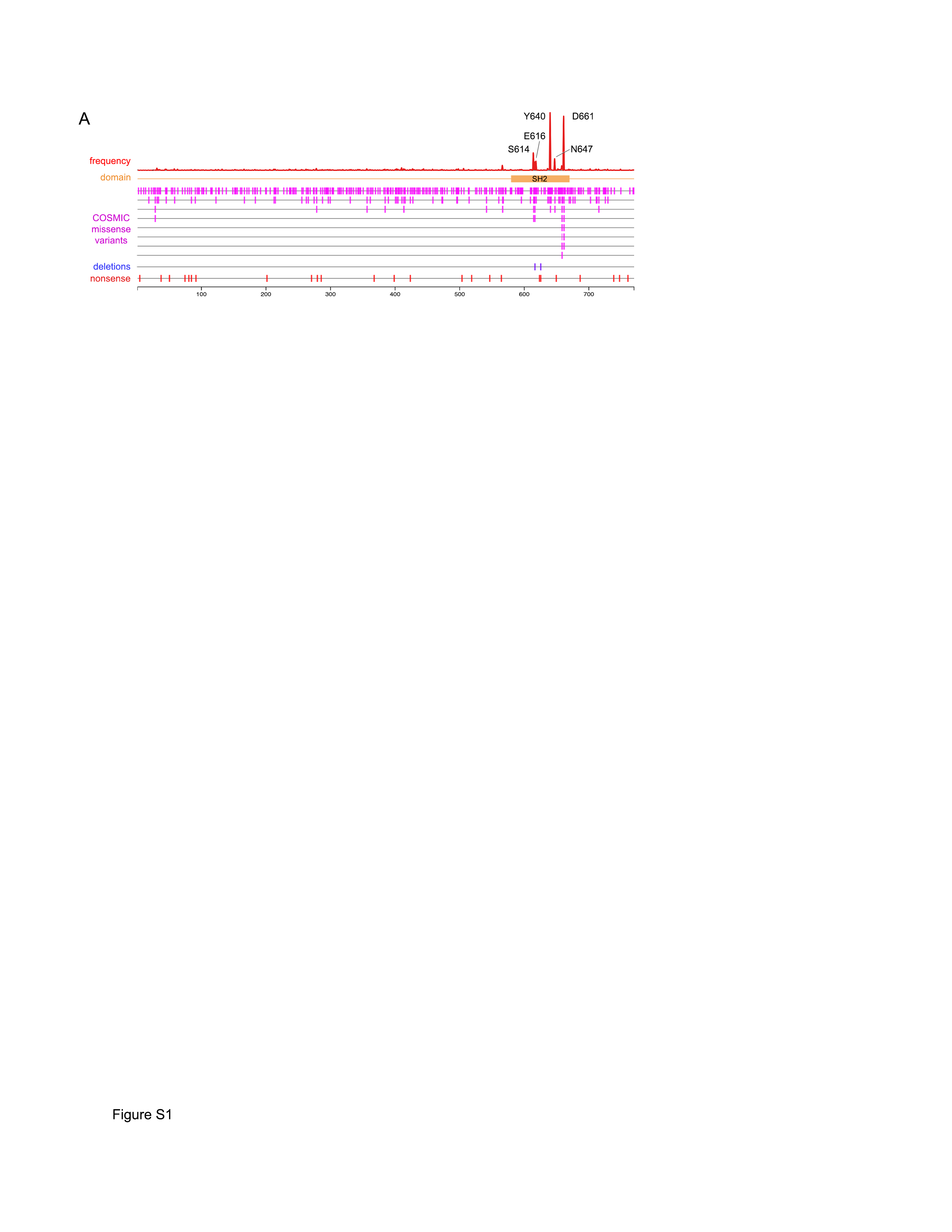

Supplement: Supplementary Figure 1 — Frequency and position of STAT3 SH2 variants. (A) Histogram shows position (x axis) and frequency (y axis) of single amino acid STAT3 variants linked to hematologic and lymphoid malignancies. Top 5 most frequent variants are noted. Each row represents a distinct variant, including deletions and nonsense variants. Full, annotated variant roster catalogued in Supplementary Table 1. [file Image1.tif]

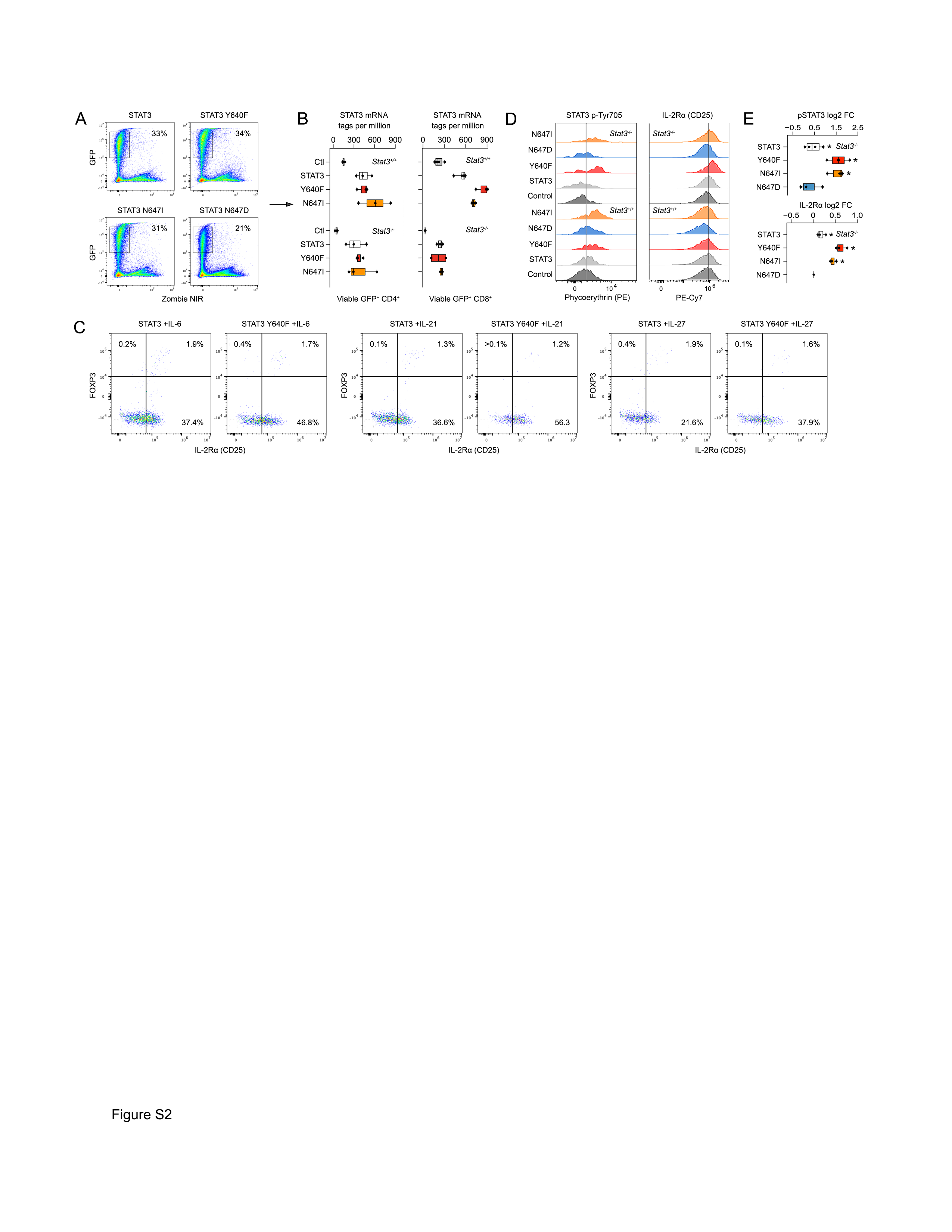

Supplement: Supplementary Figure 2 — Retrogenic system validation. (A–D) Stat3+/+ and Stat3-/- lymphocytes were transduced with control or variant STAT3 vectors, then cultured with IL-27 and assayed by cytometry or RNA-seq. (A) Cytometry pseudocolor plots show GFP expression in transduced cells. (B) Box plots show STAT3 transcript counts (TPM) in transduced CD4+ (left) or CD8+ (right) T cells. (C) Cytometry pseudocolor plots show FOXP3 and IL-2Rα in transduced Stat3-/- CD4+ T cells. (D) Cytometry histograms show p-STAT3 and IL-2Rα (CD25) in transduced Stat3+/+ and Stat3-/-CD8+ T cells.(E) Box plots compile mean fluorescence intensity values for Stat3-/- CD8+ T cells. Stars denote p< 0.05 relative to empty vector control. Replicates and statistical tests detailed in Supplementary Table 2. [file Image2.tif]

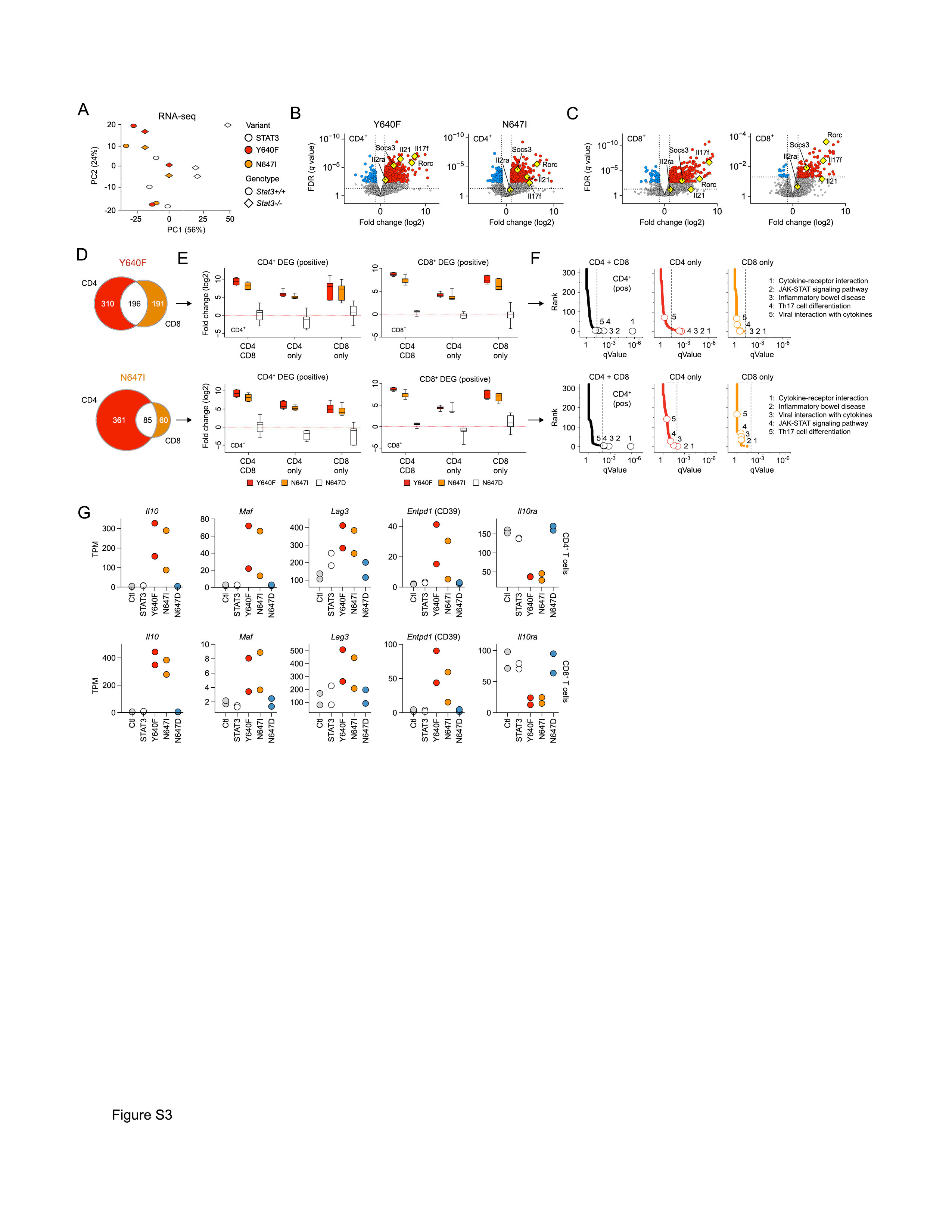

Supplement: Supplementary Figure 3 — Common and lineage-restricted effects of STAT3 SH2 variants. (A–F) Stat3+/+ and Stat3-/- T cells were transduced with control or variant STAT3 vectors, cultured with IL-27, then processed for RNA-seq (A) Scatter plot shows PCA results for CD4+ T cells. (B, C) Volcano plots show variance and log2-transformed fold change values for all transcripts captured in Stat3-/- CD4+ (B) or CD8+ (C) T cells. (D) Venn plots compare DEG mobilized by Y640F (top) or N647I (bottom) in CD4+ versus CD8+ T cells. (E) DEG were categorized based on whether mobilized in CD4+ T cells, CD8+ T cells or both (per adjacent Venn plots). Box plots compile log2-transformed, fold-change values for each category. (F) Positively regulated DEG from each category were subjected to hypergeometric testing against the KEGG database. Rankline plots shows adjusted p values and p value ranks for all pathways, with select pathways noted. (G) Scatter plots show transcript counts for Tr1-associated genes in CD4+ or CD8+ T cells. All genesets detailed in Supplementary Tables 3, 4. RNA-seq datasets further detailed in Supplementary Tables 5–7. Replicates and statistical tests detailed in Supplementary Table 2. [file Image3.tif]

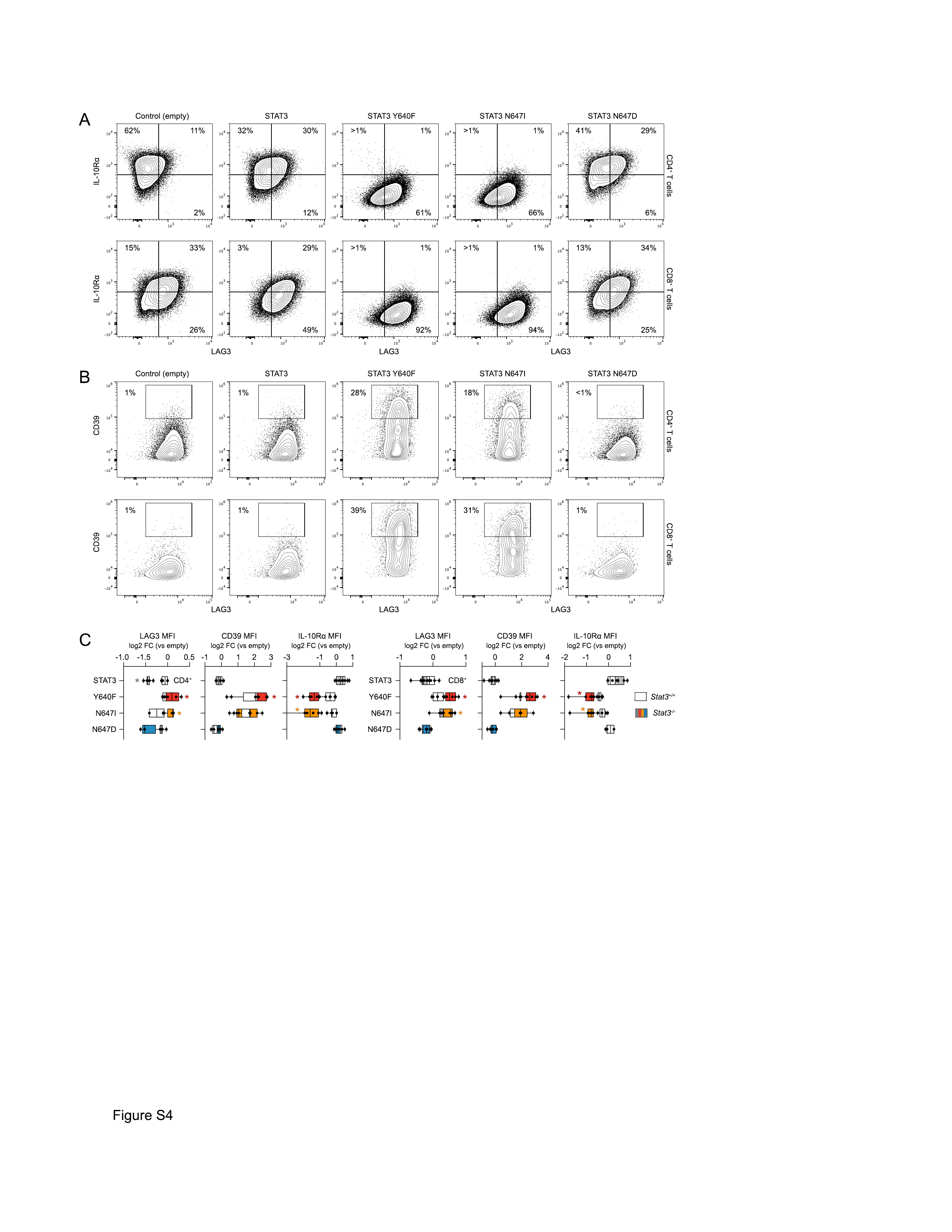

Supplement: Supplementary Figure 4 — Evidence for competition between normal STAT3 and SH2 variants. (A–D) Stat3+/+ and Stat3-/- lymphocytes were transduced with control or variant STAT3 vectors, then cultured with IL-27 and assayed by cytometry. (A) Cytometry contour plots show surface protein measurements in CD4+ and CD8+ T cells. (B) Cytometry contour plots show percentage of CD39high CD4+ and CD8+ T cells. (C) Box plots compile log2 fold change values for mean fluorescence intensities across cytometry experiments. Open boxes are Stat3+/+ cells, shaded boxes are Stat3-/- cells. Stars denote significant differences across genotypes. Replicates and statistical tests detailed in Supplementary Table 2. [file Image4.tif]

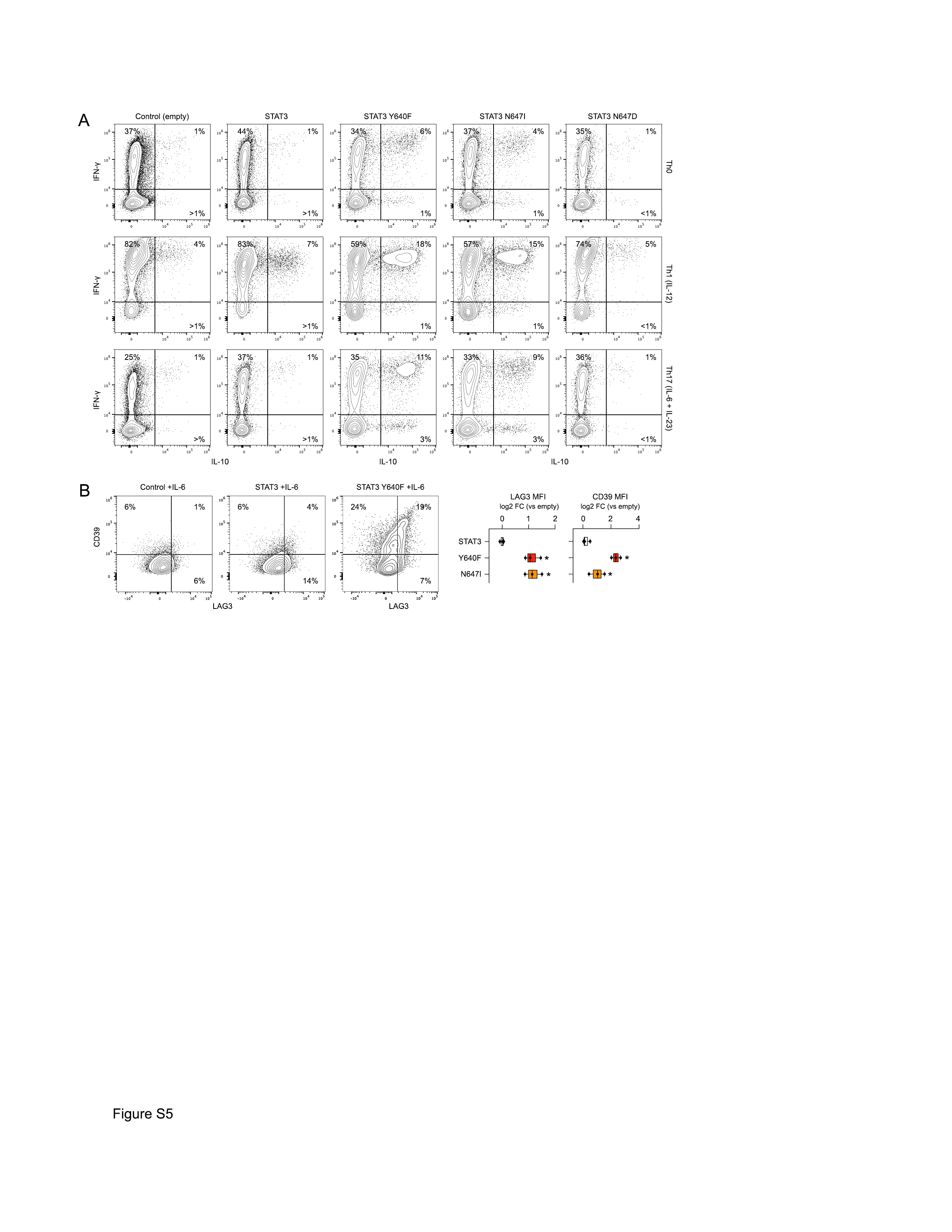

Supplement: Supplementary Figure 5 — STAT3 SH2 variants invoke Tr1 responses downstream of multiple cytokines. (A, B) Stat3-/- lymphocytes were transduced with control or variant STAT3 vectors, then cultured and assayed by cytometry. (A) Contour plots show cytokine measurements for CD4+ T cells cultured under non-polarizing, Th1 polarizing or Th17 polarizing conditions. (B) Contour plots show surface protein measurements for CD4+ T cells cultured with IL-6. Box plots compile log2 fold change values for mean fluorescence intensities across cytometry experiments. Stars denote p < 0.05 relative to empty vector control. Replicates and statistical tests detailed in Supplementary Table 2. [file Image5.tif]

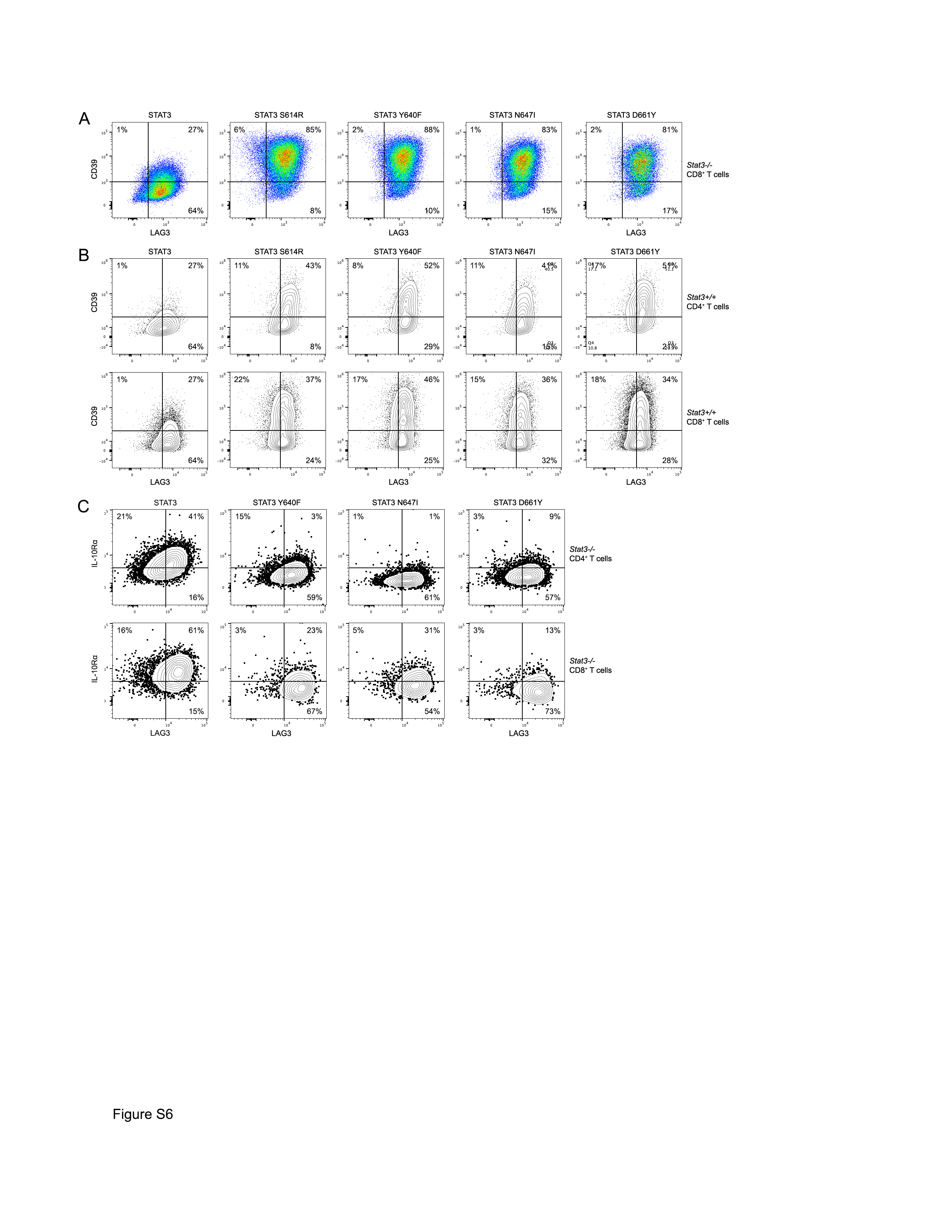

Supplement: Supplementary Figure 6 — STAT3 SH2 variants impose a Tr1 phenotype in both CD4+ and CD8+ T cells. (A-C) Stat3+/+ and Stat3-/- lymphocytes were transduced with control or variant STAT3 vectors, then cultured with IL-27 and assayed by cytometry. (A) Pseudocolor plots show LAG3 and CD39 measurements in Stat3-/- CD8+ T cells. (B) Contour plots show LAG3 and CD39 measurements in Stat3+/+ CD4+ and CD8+ T cells. (C) Contour plots show LAG3 and IL-10R⍺ measurements in Stat3-/- CD4+ and CD8+ T cells. Replicates and statistical tests detailed in Supplementary Table 2. [file Image6.tif]

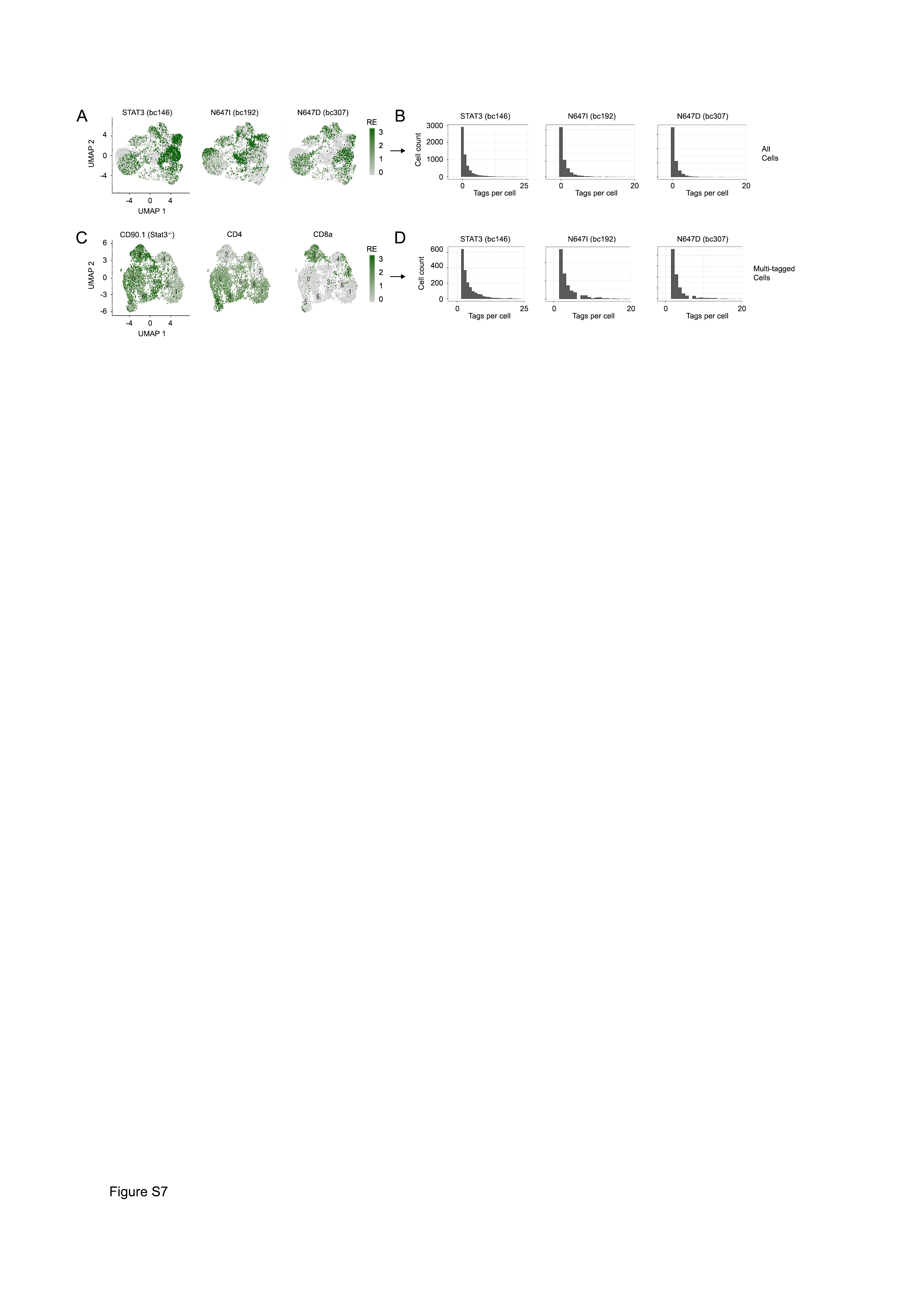

Supplement: Supplementary Figure 7 — Selection and analysis of barcode tagged T cells. (A–C) WT and Stat3-/- T cells were transduced with barcoded control STAT3, STAT3 N647I or STAT3 N647D vectors, then cultured with IL-27 and assayed by scRNA-seq (as in Figure 5A). Featureplots show distribution of barcode tags for all cells. (B) Histograms stratify cells based on number of tags detected per cell (x axis). (C) Featureplots show distribution of Cd4, Cd8a and CD90.1, which marks Stat3-/- cells, in cells bearing >1 exclusive tag (multi-tagged cells). (D) Histograms stratify cells based on the number of tags detected per cell (x axis). Only multi-tag selected cells shown. [file Image7.tif]

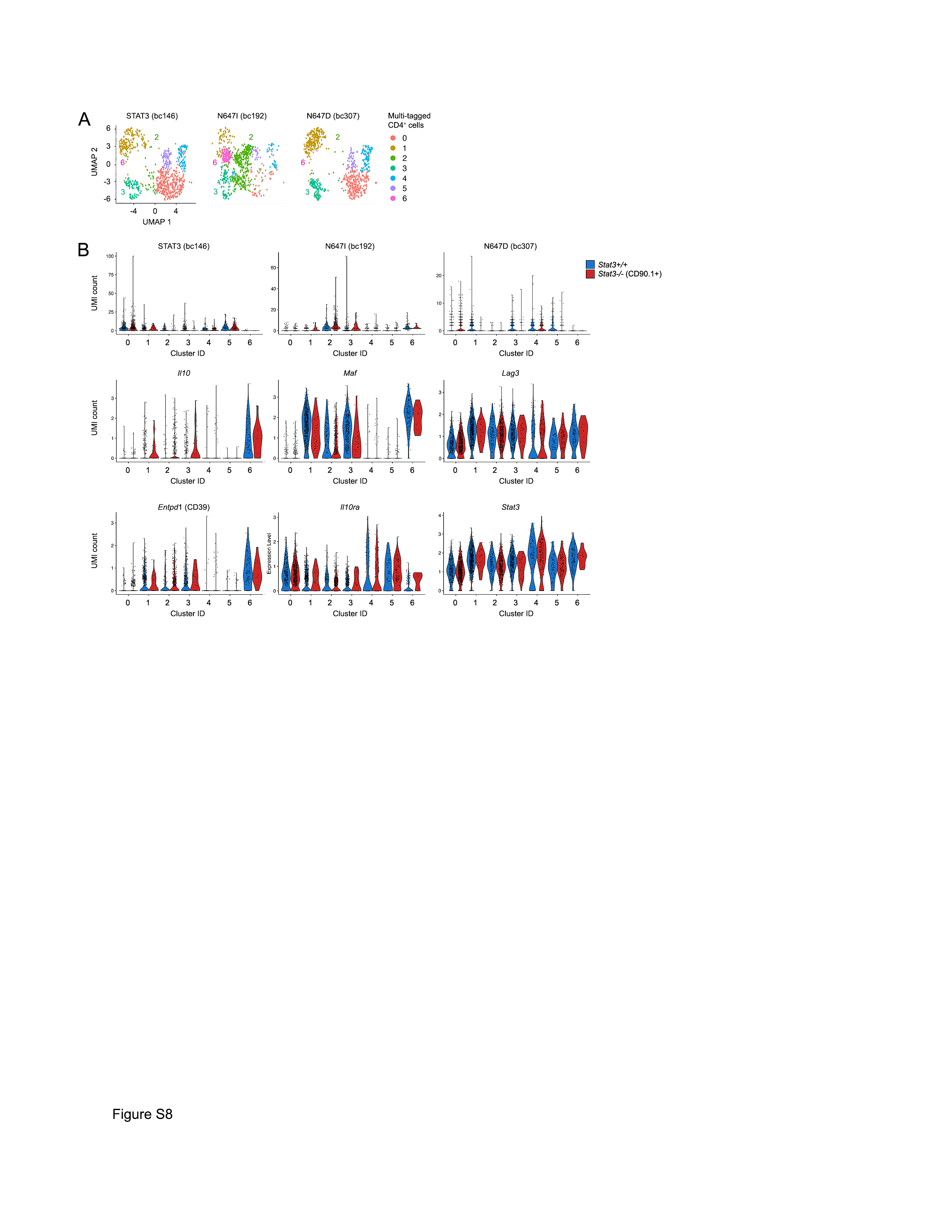

Supplement: Supplementary Figure 8 — STAT3 N647I elicits a Tr1 program in CD4+ T cells. (A, B) scRNAseq was performed and multi-tagged cells subset as in Supplementary Figure 7. (A) Featureplots show UMAP clustering of Cd4+ cells broken down by DNA tags. (B) Violin plots show UMI counts for select Tr1-associated genes in Cd4+ cells, broken down by UMAP cluster (x axis) and genotype (color). [file Image8.tif]

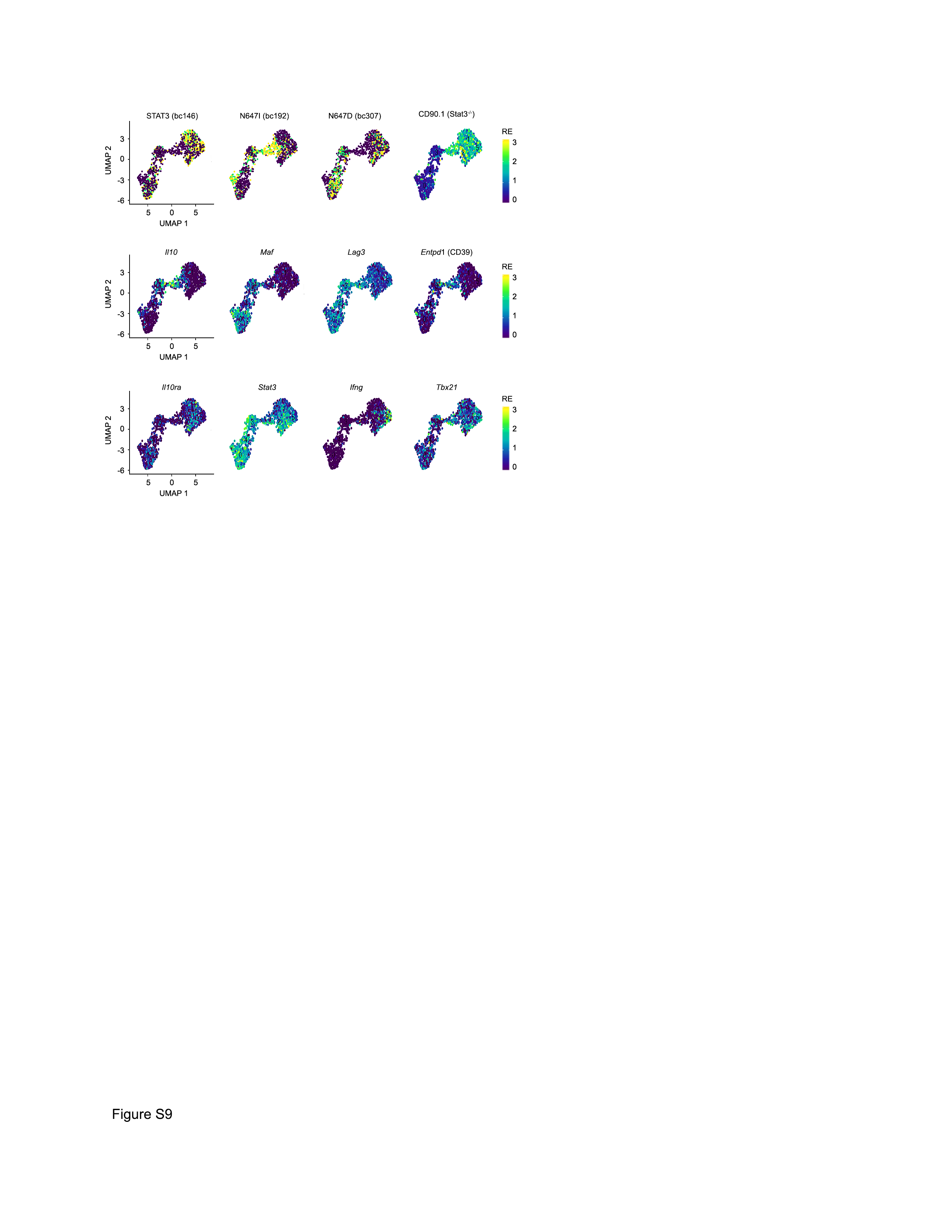

Supplement: Supplementary Figure 9 — STAT3 N647I elicits a Tr1 program in CD8+ T cells. (A, B) scRNAseq was performed and multi-tagged cells subset as in Supplementary Figure 7. (A) Featureplots show relative expression of variant-associated tags, CD90.1 and select Tr1-associated genes in Cd8a+ cells. [file Image9.tif]

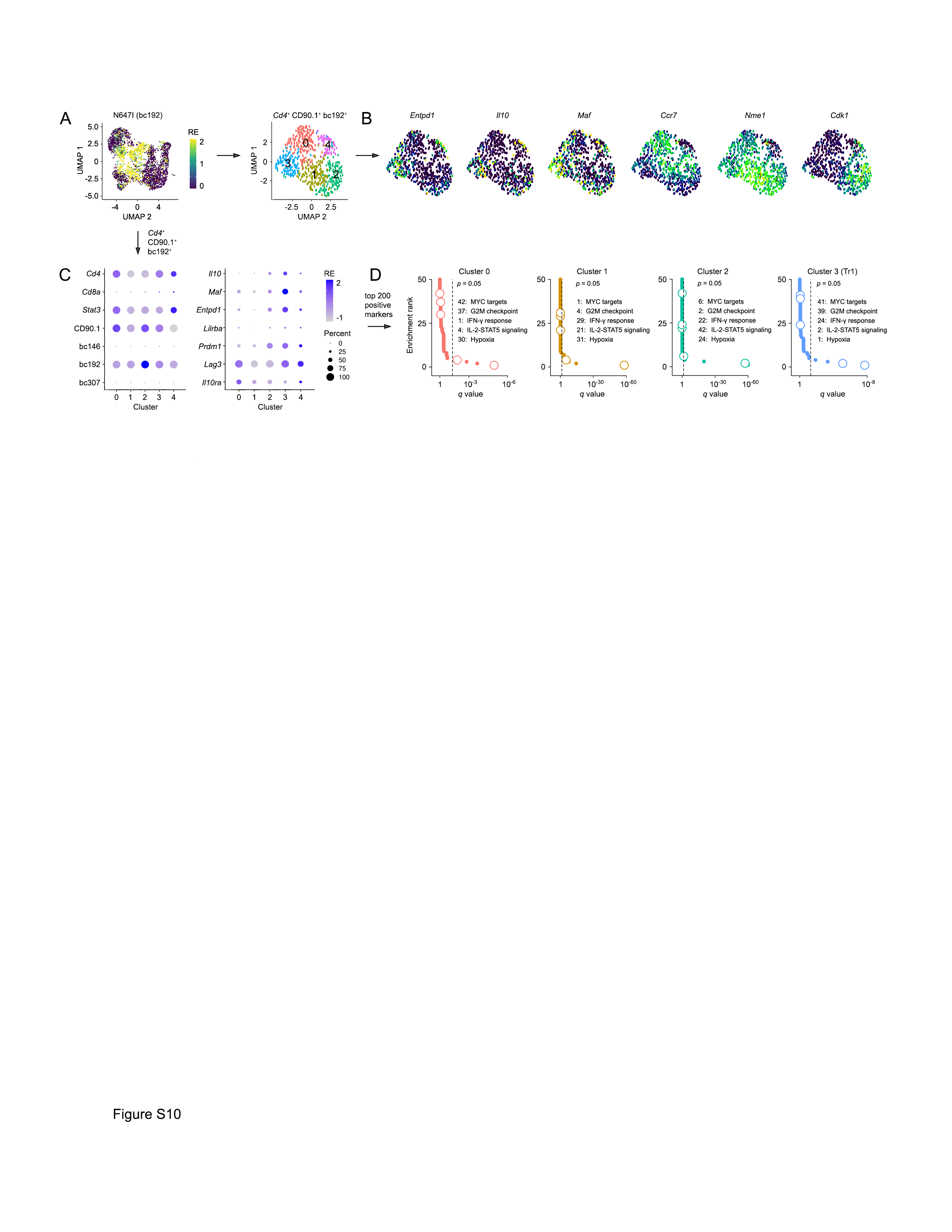

Supplement: Supplementary Figure 10 — Transcriptional heterogeneity in CD4+ T cells expressing N647I. (A–D) scRNAseq was performed and multi-tagged cells subset as in Supplementary Figure 7. (A) Left featureplot shows enrichment of DNA barcode 192, which marks cells transduced with N647I. Right featureplot shows UMAP clustering of Cd4+ CD90.1+ (Stat3-/-) barcode 192+ cells. (B) Featureplots show enrichment of markers representing each major UMAP cluster. (C) Scatter plots show relative enrichment (RE) and percent positive cells for select markers. (D) Top 200 positively regulated markers for each cluster were subjected to hypergeometric testing against the molecular signatures database. Rankline plots shows p values and p value ranks for all pathways, with select pathways and ranks noted. [file Image10.tif]

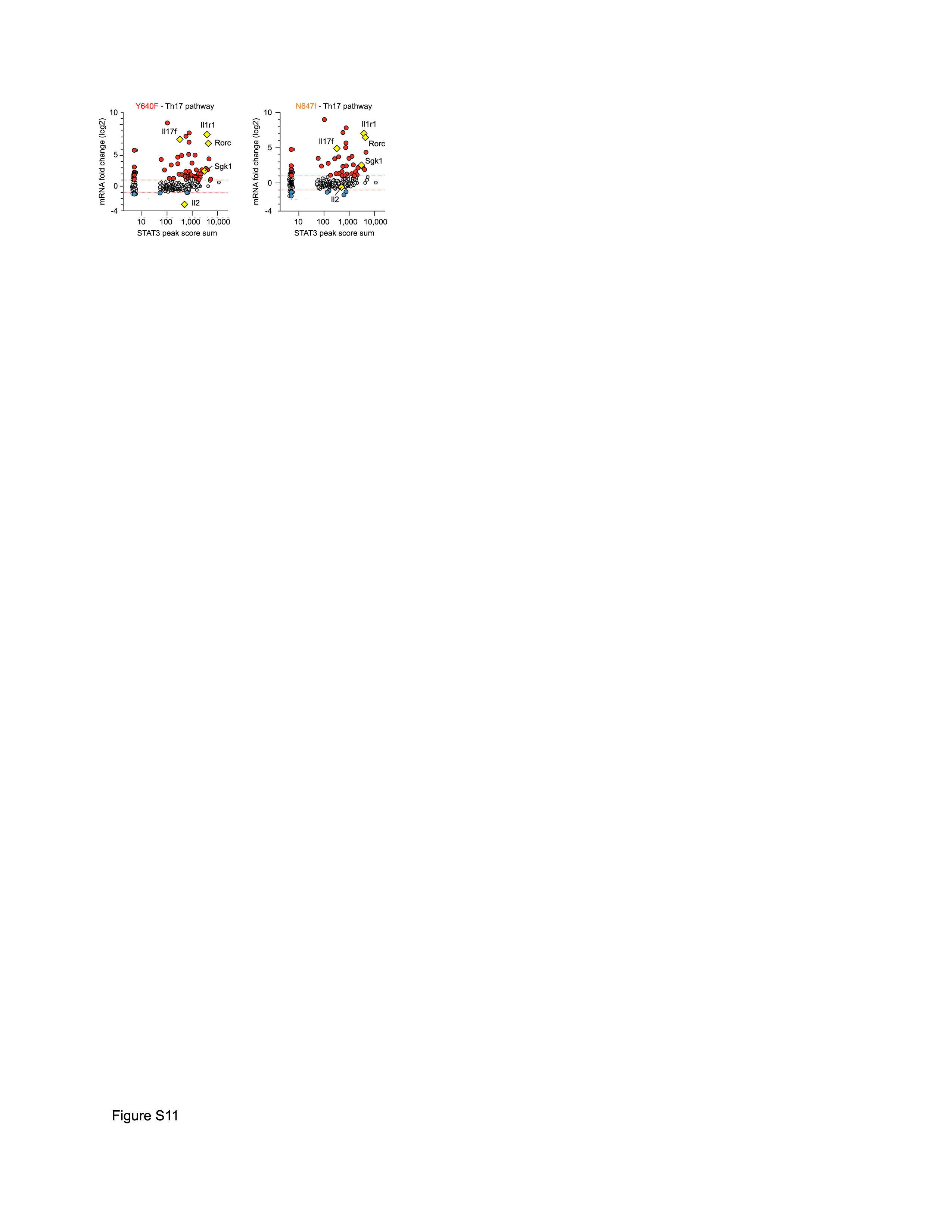

Supplement: Supplementary Figure 11 — STAT3 localizes to Th17-associated gene loci. (A) CD4+ T cell DEG (Figure 2B) were cross-referenced with a STAT3 ChIP-seq dataset captured in CD4+ T cells pulsed with IL-27. (A) Scatter plots show cumulative STAT3 peak amplitude (x axis) and transcript fold change values (normal versus variant) for Th17-associated genes. Full, annotated STAT3 peak set presented in Supplementary Table 9. Th17 geneset detailed in Supplementary Table 3. Dotted red lines denote 2-fold change. [file Image11.tif]

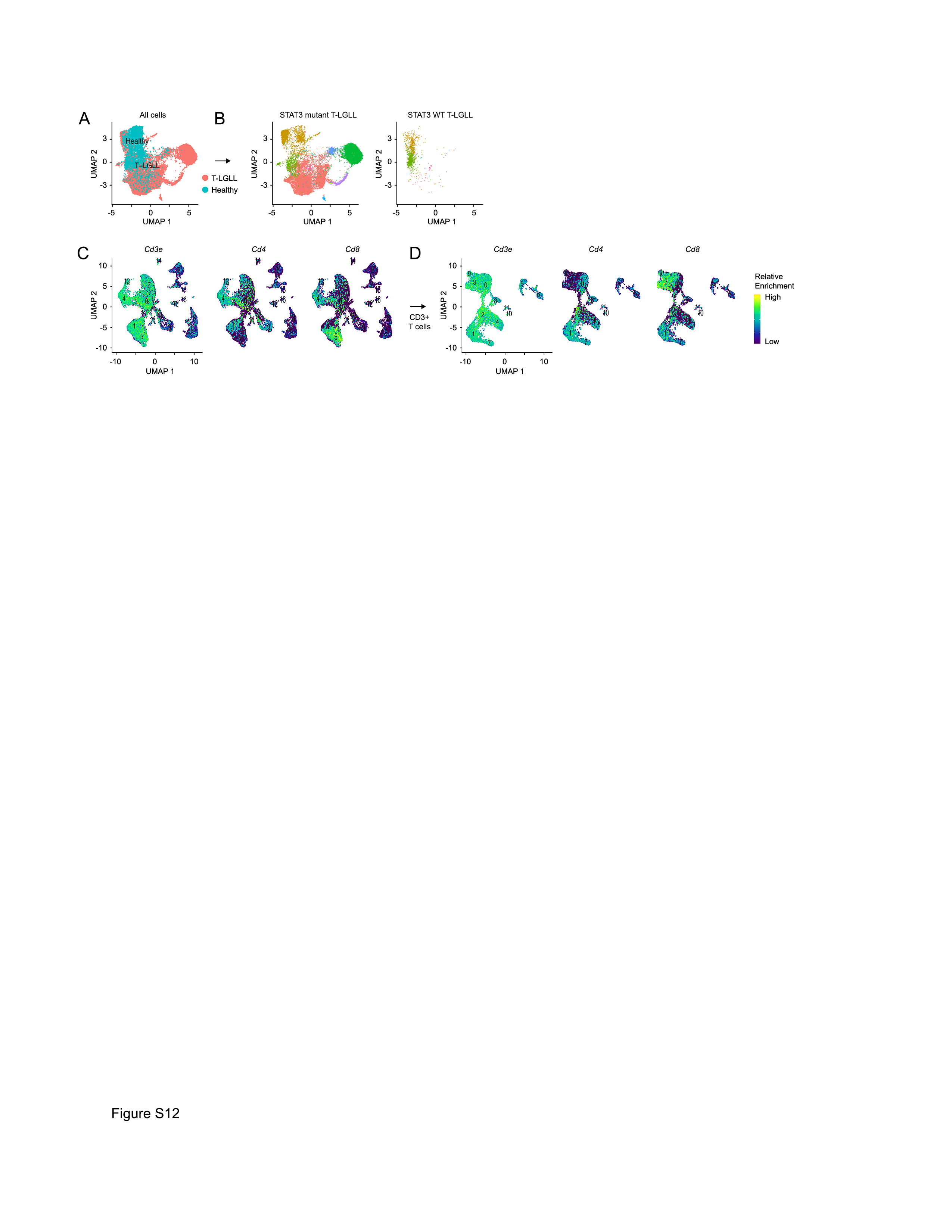

Supplement: Supplementary Figure 12 — scRNA-seq annotation and subsetting for T-LGLL and ATLL patient samples. scRNA-seq was performed on T-LGLL (A, B) and ATLL (C, D) blood samples per Huuhtanen et al. and Joo et al. (A) Featureplots show distribution of hyper-expanded T cells originating from T-LGLL patients or healthy controls. (B) Featureplots show distribution of hyper-expanded T cells from T-LGLL patients bearing mutant STAT3. (C) Featureplots show relative expression of CD3E, CD4 and CD8A in all cells assayed from ATLL patients. (D) Featureplots show relative expression of CD3E, CD4 and CD8A within the CD3E+ subset. [file Image12.tif]
